# Impact of Portion Control Tools on Portion Size Awareness, Choice and Intake: Systematic Review and Meta-Analysis

**DOI:** 10.3390/nu13061978

**Published:** 2021-06-09

**Authors:** M. Angeles Vargas-Alvarez, Santiago Navas-Carretero, Luigi Palla, J. Alfredo Martínez, Eva Almiron-Roig

**Affiliations:** 1 Center for Nutrition Research, University of Navarra, 31008 Pamplona, Spain; mvargas.1@alumni.unav.es (M.A.V.-A.); snavas@unav.es (S.N.-C.); 2 Department of Nutrition, Food Science and Physiology, Faculty of Pharmacy and Nutrition, University of Navarra, 31008 Pamplona, Spain; jalfmtz@unav.es; 3 Navarra Institute for Health Research (IdiSNa), 31008 Pamplona, Spain; 4 CIBERobn, Obesity and Nutrition, Instituto de Salud Carlos III, 28029 Madrid, Spain; 5 Department of Public Health and Infectious Diseases, University of Rome La Sapienza, 00185 Rome, Italy; luigi.palla@uniroma1.it; 6 Department of Medical Statistics, London School of Hygiene and Tropical Medicine, London WC1E 7HT, UK

**Keywords:** portion size, portion control tool, portion size awareness, tableware, weight loss

## Abstract

Portion control utensils and reduced size tableware amongst other tools, have the potential to guide portion size intake but their effectiveness remains controversial. This review evaluated the breadth and effectiveness of existing portion control tools on learning/awareness of appropriate portion sizes (PS), PS choice, and PS consumption. Additional outcomes were energy intake and weight loss. Published records between 2006–2020 *(n* = 1241) were identified from PubMed and WoS, and 36 publications comparing the impact of portion control tools on awareness (*n* = 7 studies), selection/choice (*n* = 14), intake plus related measures (*n* = 21) and weight status (*n* = 9) were analyzed. Non-tableware tools included cooking utensils, educational aids and computerized applications. Tableware included mostly reduced-size and portion control/calibrated crockery/cutlery. Overall, 55% of studies reported a significant impact of using a tool (typically smaller bowl, fork or glass; or calibrated plate). A meta-analysis of 28 articles confirmed an overall effect of tool on food intake (*d* = –0.22; 95%CI: –0.38, –0.06; 21 comparisons), mostly driven by combinations of reduced-size bowls and spoons decreasing serving sizes (*d* = –0.48; 95%CI: –0.72, –0.24; 8 comparisons) and consumed amounts/energy (*d* = –0.22; 95%CI: –0.39, –0.05, 9 comparisons), but not by reduced-size plates (*d* = –0.03; 95%CI: –0.12, 0.06, 7 comparisons). Portion control tools marginally induced weight loss (*d* = –0.20; 95%CI: –0.37, –0.03; 9 comparisons), especially driven by calibrated tableware. No impact was detected on PS awareness; however, few studies quantified this outcome. Specific portion control tools may be helpful as potentially effective instruments for inclusion as part of weight loss interventions. Reduced size plates per se may not be as effective as previously suggested.

## 1. Introduction

Large portion sizes (PS) increase consumption, and eating smaller portions is recommended as a weight control strategy [1,2,3]. However, many people report difficulties enacting this advice [4]. While wider changes in the food environment may be needed to impact on portion control at the population level, some food- and individual-level strategies have shown promise, including the use of portion-controlled meals, reduced pack sizes, modified tableware, attentive eating and portion control strategies as part of weight management programmes [5]. Traditionally, educational aids to guide portion sizes have been used as part of such programmes; however, most of these aids are image or text-based [6,7,8,9,10] and deemed to be of limited effect [11,12], in part due to inconsistent portion size standards [13,14]. Recently, three-dimensional portion control tools have been commercialised, with claims to control portion sizes by either physically delineating volume (i.e., portion pots [15], guided tableware [16]) or by including visual prompts for appropriate amounts, such as calibration marks in tableware and serving utensils [16,17]. Such instruments, being practical in nature, have the added potential to be a useful strategy in an environment where large portions are often the norm [18,19]. For example, impulsivity and perceptions of how much is appropriate to eat are factors that could mediate certain people’s intention to consume larger or smaller portions in such environments [20]. Portion control tools may help to modulate these factors by promoting meal planning and “correcting” misperception of inappropriate portion sizes at the time of serving [21,22]. However, not all commercially available portion control tools have been demonstrated to be scientifically valid, and much controversy exists over the real impact of reduced size tableware, including for solid food [23,24,25,26,27] and drinks [28,29,30,31].

Up to now, the two largest meta-analyses (MA) exploring the role of portion control tools on food intake considered the use of such tools alongside other portion control strategies (i.e., Hollands et al. and Zlatevska et al. MA [18,32] used also packaging and PS offerings). Additional weight management strategies such as dietetic counselling were included in some studies also, making it difficult to determine the effect of PS tools per se. Two other previous MA specifically looking at portion control tools [23,24] focused only on a type of tableware (i.e., small vs. large plates and bowls) and are now slightly outdated (2014, 2016). These MA however showed important influences of study design not necessarily accounted for in earlier analyses (i.e., who serves the food and what the volunteers know about the study); therefore, new reviews need to integrate these factors as covariates.

The present review takes a comprehensive approach to include all portion control tools available until present and quantitatively compares them on their impact on portion size learning or awareness, choice and intake when data are available. For example, educational aids and PS estimation tools that provide direct feedback are also included as a new aspect on PS control. We include also data on user experiences and acceptance of portion control tools, which are less frequently reported.

Based on a recent umbrella review [5], tableware appeared as a promising strategy to control portion sizes at the individual level, so the present work looks specifically into this portion tool category. Tableware is a heterogeneous group though (including not just differently sized plates, bowls and cutlery but also tools of specific design carrying for example calibration marks, sectors, images, etc.) Therefore, different types of tableware need to be considered so specific tool designs with potentially significant impact can be identified.

Overall, the evidence demonstrating the potential role of portion control tools on portion size control and weight management is either inconclusive or still limited for specific types of instruments. Based on the current literature gaps, the aim of this work was to conduct a systematic review of the literature to (a) describe the range and effectiveness of existing portion control tools for foods and drinks and (b) quantify the effects of such tools on learning/awareness of appropriate portion sizes, portion size choice and portion size consumption, in adults and children, using a meta-analytic approach.

## 2. Materials and Methods

This systematic review and meta-analysis was performed following the Cochrane Guidelines for Systematic Reviews and PRISMA reporting recommendations [33,34]. The review was registered in the International Prospective Register of Systematic Reviews (PROSPERO) with registration ID CRD42020200775.

### 2.1. Search Strategy

Searches were conducted on PubMed and Web of Science (WoS) in July 2018 and later again in February 2019 and subsequently updated during July 2020. Searches were complemented with an internal database of publications (years 1989–2020) and with Google for non-validated tools and grey literature. Restrictions included date of publication between January 2006 and July 2020, Humans and English or Spanish language. The bibliographic list of a PhD thesis on portion size and energy intakes [35] was also title-screened. Further titles were identified by cross-referencing from all these sources and from the authors’ knowledge.

Articles were identified using various combinations of the following key words: tableware, dishware, portion control, portion size, calibrated, tools and software. Search equations were used to find the relevant articles in both databases (see Supplementary Materials for details of search strategy).

### 2.2. Eligibility Criteria

Studies were selected for review using population, intervention, comparison group, outcome, and study design (PICOS) criteria (Table 1).

To be included in the review, records needed to report on the use of a tool or instrument (validated or not) to control food and drink portion sizes (including PS estimation aids when reference can be made to recommended portion sizes) and that did not require significant input from a health professional in the form of clinical guidance, i.e., via regular counseling. That is, the tool should provide some sort of immediate feedback to the user to enable them to use it appropriately with minimal professional input. Tools could include utensils of reduced capacity (i.e., smaller plates or bowls) and of specific design (i.e., calibrated/portion control plates, bowls and glasses), technologies (software or websites), image-based educational guides, cooking utensils (i.e., measuring pots and spoons), and serving tools (serving spoons and trays). Web pages and other sources describing existing and accessible instruments, tools or software were only included if the tools met the above inclusion criteria. Instruments to control portion size for coffee, tea, diet drinks and decaffeinated drinks were included as managing the intake of these products may be of interest for some individuals in order to reduce intake of certain compounds such as caffeine, sweeteners or acidic compounds. Monitoring of water intake may be desirable in certain conditions and for healthy hydration, so portion control tools for water were also included.

Discontinued tools (i.e., no longer accessible from the manufacturer) were excluded. Packaging-based portion control strategies, including snack box sizes were also excluded as these represent a wider environmental strategy that falls beyond the scope of this work. Condiments provide minimal energy, so tools related to condiments were also excluded.

### 2.3. Procedure for Selection of Studies

Two authors performed the searches (MAVA and SNC) and conducted the title and abstract screening. A third author (EAR) solved divergences with the first authors, plus independently screened 10% of the titles and abstracts.

**Figure 1:**
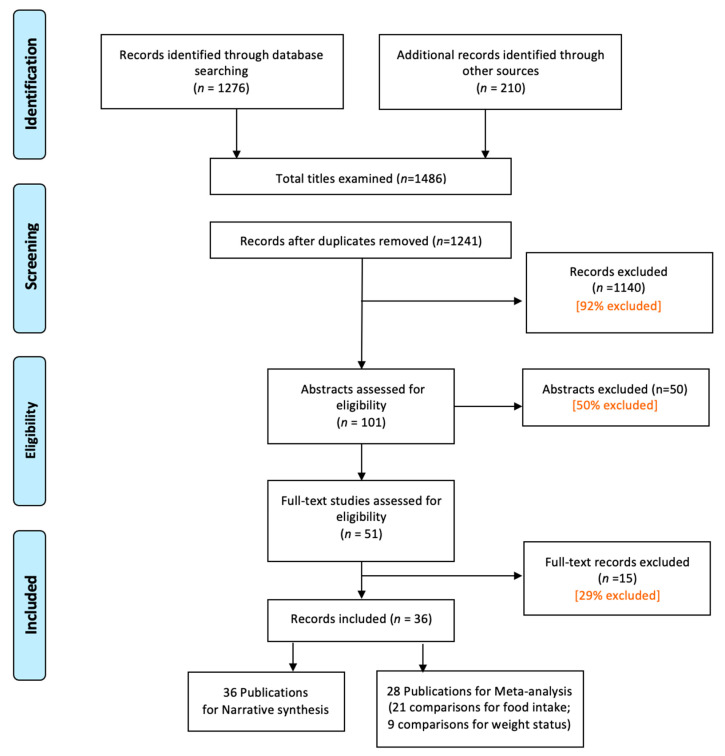
PRISMA Flow diagram for study inclusion (based on [34]).

A total of 1486 titles were identified (1276 through database searching and 210 from other sources). After removing duplicates, 1241 records were retained. Of these, 101 abstracts were assessed for eligibility, and 51 full-text articles were retrieved, resulting in a final sample of 36 eligible publications for the narrative synthesis. Of these, 28 publications were included in the meta-analysis (Figure 1).

### 2.4. Data Extraction Process

Data were extracted by two investigators (MAVA, EAR) and disagreements discussed and agreed with a third author (SNC) when necessary. A standardized data extraction form was used to collect methodological and outcome variables from each source including the following: authors, publication year, country, number of studies included in the paper, exposures to the stimuli, serving condition (self-served, served by others, both), type of manipulation, study design, study duration, nature and number of tools used, main outcome measures, sample size, sex, age and BMI. We contacted corresponding authors for missing or additional information when necessary.

Main outcome measures included portion size awareness (knowledge or learning), portion size choice (selection) and portion size intake (consumption)**,** for the whole meal and/or meal components. Other outcome data included: tool-related perceptions/experiences (e.g., acceptance, usability, perceived efficacy), biochemical (glucose, HDL, etc) and anthropometric (weight, BMI, waist and hip circumference, etc.) markers, portion-size perceptions and eating context (i.e., acquaintance). For studies reporting specific macronutrients or foods, we analysed the impact of the tool on selected and/or consumed amounts for vegetables, protein, carbohydrates and fat. Based on previous reviews suggesting a potential role for specific co-variates [24], we also extracted data on subject awareness of the study purpose, strategy used (only the tool vs. dietitian/other strategy involved) and format of administration (self-served vs. fixed portion size).

The following operational terms were used in relation to outcome measures:

- Portion control tool: an instrument that provides direct feedback to the user on how much to serve or consume, including 3D tools, 2D educational aids and/or technology-based tools.
- Portion size selection: amounts selected (in g, mL or kcal/kJ).
- Portion size consumption: amounts consumed (in g, mL or kcal/kJ) or proxy measures (e.g., % sales).
- Portion size awareness or learning of appropriate portion sizes: ability to judge what is an appropriate amount to consume of a particular food/beverage depending on individual needs at the time of consumption (i.e., for healthy eating, weight management or other therapeutic purposes). Awareness may be reported as the percentage (%) error in estimation vs. the actual amounts, calculated as the difference between actual and estimated amounts, relative to the actual amount. Actual amounts may be reported in volume/weight/cm/other standard unit of measurement. Estimated amounts may be measured in household measures, categorical size estimates (small, medium, large), photographs or other systems and converted into standard units of measurement.

For the meta-analysis, mean and standard deviation (SD) data were extracted when available to calculate effect sizes for the main outcomes including change in portion size awareness, choice and intake; BMI change; and body weight change. When numerical data were not reported or were unclear, the authors were contacted for information for articles less than 10 years old.

### 2.5. Risk of Bias and Quality Assessment

As part of the quality assessment, computed effect sizes were contrasted with data published in related reviews [5,18,23,24,32] and in its absence, by duplicate manual computation.

Risk of bias (ROB) was explored using an adaptation of the Cochrane ROB guidelines [33,36], suitable for behavioral studies. Items assessed included the use of blinded participants, potential confounding variables and methodological limitations. ROB evaluation was carried out in duplicate by three independent authors (EAR, MAVA and SNC). The results were compared between a pair of evaluators, and in case of discrepancy, a third author was involved to reach consensus.

Publication bias and other sources of heterogeneity were explored via funnel plot asymmetry when sufficient studies were available [37]. Asymmetry was further tested by Egger´s test using both random and fixed effects MA [38]. The I^2^ heterogeneity index was used as a measure of the inconsistency of the effect estimates between comparisons across different studies/comparisons (low heterogeneity was indicated by I^2^ = 25%, medium I^2^ = 50%, high I^2^ = 75%) [39].

### 2.6. Data Management and Statistical Analyses

Data from all eligible sources were compiled in an internal database and are presented by outcome measure in the tables and text, plus in the Supplementary Information.

For the MA, continuous outcomes are summarized by effect size, calculated as the standardized mean difference (SMD) or Cohen´s *d*. This metric reflects how much less is chosen or consumed with a smaller, calibrated or specially designed portion size tool vs. a larger, plain or “control” tool/condition. A negative value for the SMD/Cohen’s *d* reflects a desired impact of the intervention tool (i.e., smaller portion size chosen or consumed), with a larger mean difference reflecting a larger effect. For portion size awareness, a negative value for SMD/Cohen´s *d* reflects no impact of the tool on portion size learning or awareness. Magnitude of the effect size was based on Cohen’s *d* criteria [40]: *d* ≤ 0.2 small; 0.2 *d* 0.8 medium; *d* ≥ 0.8 large.

A database was generated from the articles that reported the necessary quantitative data for calculating the effect size. For each article, we calculated the SMD and the standard error of the SMD (SeSMD) between conditions via generic inverse variance and applied this model in STATA v16.0 (StataCorp LLC, Texas, USA) using the Meta command with the DerSimonian and Laird model [41]. We chose SMDs because despite the type of outcome being the same across the subset of studies, such outcome may be reported in different scales, i.e., food intake may be reported in grams (g), kilojoules (kJ) or calories (kcal). To calculate the SMD, we used two different formulae based on the nature of the data, as previously reported [23] and based on current recommendations [33,42,43].

For studies with a between-subjects (parallel arms) design we used

(1) $$\mathrm{SMD}=\frac{\mathrm{Mean}\;1-\mathrm{Mean}\;2}{\mathrm{SDdiff}}$$

where Mean 1 and Mean 2 are the mean of the comparisons of the smaller or control tool vs. control condition, respectively. SDdiff is the SD of the difference between the means.

For studies with a within-subjects design (crossover) we applied an adjustment for correlation between conditions:

(2) $$\mathrm{SMD}=\left(\frac{\mathrm{Mean}\;1-\mathrm{Mean}\;2}{\mathrm{SDdiff}}\right)*\left(\sqrt{2\;\times \;\left(1-r\right)}\;\right)$$

where Mean 1 and Mean 2 are the mean of the smaller or control tool vs. control condition, respectively; SDdiff is the SD of the difference between the means and r is the within-subject correlation which we estimateas 0.8, based on data compiled in Robinson et al. 2014 [23], showing that such correlation in this type of crossover studies ranges between 0.76 and 0.93. Details of computation of the standardized deviation difference (SDdiff) are given in the Supplementary Material.

The above final correction factor ($\sqrt{2\;\times \;\left(1-r\right)}$, assumes constant variance across repeated measures, and was applied as recommended by Lakens [43], using r = 0.8 [23]. SMDs are reported as Cohen´s *d* in the text.

For crossover studies, if the same subjects were exposed to more than two conditions, to avoid redundant comparisons, we excluded the intermediate comparison (i.e., if the study used three different plate sizes: small, medium and large, we calculated the SMD of the small plate vs. the large plate, excluding the medium size plate). Same criteria were applied on interventions with three or more groups, as differences between intermediate conditions are typically of smaller magnitude [23,44]. For crossover studies where portion size was manipulated alongside tool type [45,46], the smaller portion size condition was chosen for inclusion in the MA to mitigate the portion-size effect and a confirmatory sensitivity analysis was conducted with the larger portion size condition. When it was not possible to separate the effect of the tool from that of the portion size, analyses were repeated with and without that specific study. All comparisons were computed in the same direction, i.e., small vs. large tool or control vs. intervention, and were graphically displayed using forest plots. Subgroup analyses by tool type were carried out when there were sufficient data (i.e., ≥2 comparisons per group). We conducted separate analyses to identify potential influences of subject awareness of the study purpose, strategy used (only the tool vs. other strategy involved) and format of administration (self-served vs. fixed PS). Study duration was also examined for the studies reporting weight status variables. Separate forest plots were produced for studies reporting weight status variables at 3 months and at 6 months. We also repeated the analysis for PS intake and PS choice after excluding one study from Cornell University out of concern of potential data biases [47]. The specific impact of tool type was explored after recoding tool type in the PS intake analysis. Thus, studies that combined plates and bowls were recoded as a new tool type reflecting specific tool influence.

## 3. Results

### 3.1. Overview of the Studies and Tools Identified

Thirty-six articles encompassing 40 interventions (referred to as “studies”) involving at least one comparison between tools were identified for this review. These included 31 RCTs (13 within-subjects and 16 between-subjects studies, and two studies combining within and between-subjects analyses), five non-RCTs, two observational studies, one qualitative study and one mega-analysis study. Thirty-one comparisons (from 28 articles) were included in the meta-analysis representing a sample of over 2903 subjects.

The studies covered four continents: America (Canada and USA), Asia (Korea and Sri Lanka and Turkey), Europe (Netherlands, Switzerland and UK) and Oceania (Australia and New Zealand). Twenty articles examined healthy adults (both sexes) with and without overweight/obesity. Five studies sampled children [46,48,49,50], one sampled children and adolescents [51], four sampled women only [52,53,54,55,56], four sampled individuals with specific conditions (pregnancy, acute coronary syndrome or type 2 diabetes) [53,57,58,59] and four studies sampled healthy adults with particular knowledge (nutrition experts, undergraduate students from Cornell University or university clerical staff) [60,61,62,63].

Mean BMI of adult study participants in 15 studies was over the healthy-weight range (overweight and obesity) while only four studies involved normal weight adults exclusively. Another eight studies included subjects with either normal weight, overweight or obesity. The remaining six papers on adults did not report the BMI range.

A summary of all studies categorized by tool type is included in Table 2.

Full details of all studies included in the review can be found in the Supplementary Table S1.

### 3.2. Range of Tools Identified

**Figure 2:**
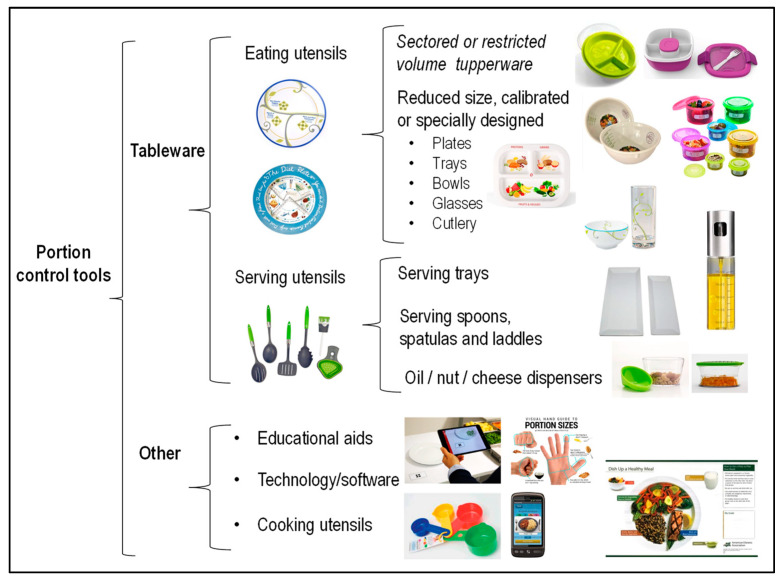
Taxonomy of available portion control tools (compiled by the authors from various sources; see text and Supplementary Table S1). Pictures of calibrated tableware, portion control pots and hands poster kindly shared by Precise Portions NLS [16], TheDietPlate.com [79], GreatIdeasInNutrition.com [80], Rosemary Conley En-terprises [15] and Flexible Dieting Lifestyles LLC [81] respectively. Picture of ServAR tablet application from [68]. We have sought to obtain permission to reproduce published images from all providers.

A wide variety of instruments were identified from the database searches and were categorized through a taxonomy of tools including 5 clusters (Figure 2). Clusters 1–2 encompass all tableware and consist of eating/drinking utensils and serving utensils. Clusters 3–5 include other tools that are not tableware such as educational aids, computerized tools and cooking utensils. In addition, grey literature searches (Google and related platforms) identified a number of commercial products claiming to help control portion sizes for both adults and children. Examples include divided trays or plates with sectors, cheese graters, oil dispensers, nut and salad dressing containers and divided lunch boxes. Most of these products lacked scientific evidence and so these tools were not investigated further. Portion control sets for which published data exists have been included in the analyses.

Eating/drinking utensils included differently sized, calibrated or specially designed Tupperware and crockery/glass or tableware made with other material (e.g., plant-based plates, bowls, glasses and cups). Serving utensils included also differently sized or calibrated serving dishes/platters and serving spoons, ladles and scoops. Educational aids are all image-based tools and included pre-portioned meal diagrams, hand-based portion guides; and non-food (reference) object portion guides. Computerized tools included web or mobile applications/software for tablets and phones (see Supplementary Table S1 for detailed information).

An overall description of each paper including details of the main outcomes is presented in Table 3 (portion size awareness), Table 4 (portion size choice), Table 5 (portion size intake) and Table 6 (weight status). Further details of all publications can be found in the Supplementary Table S1.

Seven articles reported changes in portion size awareness or learning. Portion size choice was measured as served amount in 12 articles, while portion size intake was reported as energy (kcal/kJ) or amount consumed (grams, ounces, proxy measures) in 21 articles. Nine articles reported change in body weight or in BMI. The total of these articles amounts to more than 36 because some report more than one outcome measure (e.g., choice and intake, intake and weight status, awareness and intake). Articles reporting more than one relevant outcome have been included in the corresponding sections and tables, with emphasis on each specific outcome measure (see Section 3.3, Section 3.4 and Section 3.5 below).

### 3.3. Results of Studies Examining Portion Size Awareness

Seven articles included portion size awareness and learning as main study outcome (Table 3). Only one study in this category included educational aids as a tool to enhance/promote portion size knowledge [12]. The remaining studies used computerized tools (online programme and mobile phone application) as main strategy. When assessing the overall impact of the included studies (Table 2), three out of seven studies (43%) reported an impact, mainly due to interventions using online programmes.

**Figure 3:**
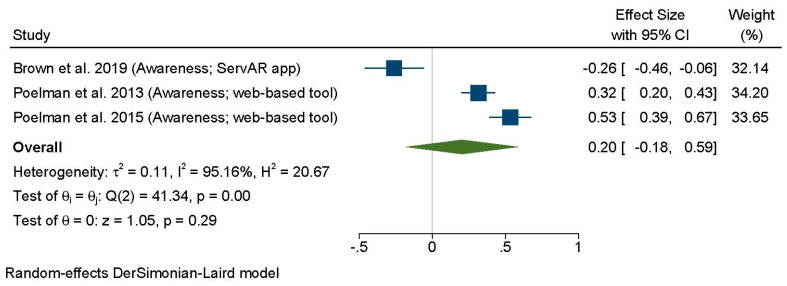
Forest plot for the analysis of all comparisons examining the effects of portion control tools on portion size awareness using random-effects meta-analysis. Contributing comparisons are represented by a filled square with horizontal lines, where the area of the square depicts the contribution of the study to the full analysis, and the horizontal lines indicate the 95% CIs for each study. Studies displaced to the left of a 0 line demonstrate a finding in favor of the tool not improving portion size learning or awareness, whereas those to the right demonstrate a finding in favor of the tool enhancing portion size learning or awareness. The diamond at the base of the plot represents the combined effect (Standardized Mean Difference) with 95% CIs.

Only three of the seven articles provided sufficient data to be included in the meta-analysis (Figure 3). This revealed that interventions using web-based or mobile applications did not have a significant impact on portion size awareness. Although some individual studies showed promising results for web-based tools, the overall effect size was small, and heterogeneity across studies was high (*d*: 0.20; 95% CI: –0.18, 0.59; I^2^ = 95.16%).

### 3.4. Results of Studies Examining Portion Size Choice

Fourteen studies (Table 4) examining the effect of portion control tool on served portion size/amount were reviewed. Most of the studies in this category used differently sized tableware (bowls and plates) as main strategy and reported substantial impact on serving sizes (12/14, 86%). Studies in this group included both children and adults with five studies performed in children [48,49,50], as a whole, reporting a significant impact on portion size choice.

**Figure 4:**
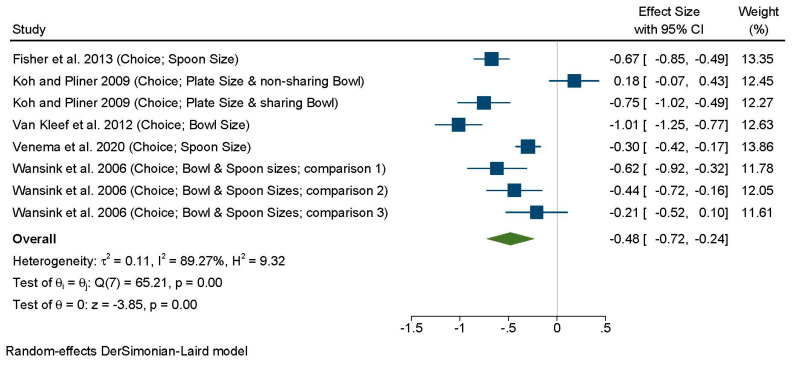
Forest plot for the analysis of all comparisons examining the effects of portion control tools on portion size choice (served amount) using random-effects meta-analysis. Contributing comparisons are represented by a filled square with horizontal lines, where the area of the square depicts the contribution of the study to the full analysis, and the horizontal lines indicate the 95% CIs for each study. Studies displaced to the left of a 0 line demonstrate a finding in favor of the portion control tool to reduce serving sizes, whereas those to the right demonstrate a finding in favor of the portion control tool promoting an increase on served amounts when compared with the control condition. The diamond at the base of the plot represents the combined effect (Standardized Mean Difference) with 95% CIs. Wansink et al. 2006 Comparison 1—Small bowl combined with small spoon vs. Large bowl combined with large spoon. Wansink et al. 2006 Comparison 2—Small bowl combined with large spoon vs. Large bowl combined with large spoon. Wansink et al. 2006 Comparison 3—Large bowl combined with small spoon vs. Large bowl combined with large spoon.

Only five of the 14 studies reported sufficient data to be included in the meta-analysis (Figure 4), encompassing eight independent comparisons. Across all tools, there was a significant effect of tool on portion size choice (*d* = –0.48; 95% CI: –0.72, –0.24; I^2^ = 89.27%). Six comparisons clearly showed a tendency for the tool to reduce serving sizes, with a medium sized effect, while for two comparisons, the reduced sized plate or bowl did not impact served amounts (Wansink et al., 2006 Comparison 3 [61] and Koh and Pliner, 2009, non-shared bowl [52]). Excluding one study where the portion tool was manipulated alongside portion size [46], did not change the results (Supplementary Figure S1).

### 3.5. Results of Studies Examining Portion Size Intake

Twenty-one studies were included in this group (Table 5). Nine of these studies (11/21, 52%) reported an impact of using a portion control tool on food portion size (either as consumed amounts or energy intake). Using a smaller plate to reduce consumed amounts was a common strategy; however, it inconsistently resulted in reduced intake when the plate was used on its own (i.e., without a reduced size bowl or cutlery). Smaller plates were only effective when combined with smaller bowls in a short-term school intervention [50] and when combined with a shared serving bowl (as opposed to non-shared) in a controlled study [52].

Smaller bowls alone were effective as part of a weight loss intervention in adults with diabetes [53]), but not in two controlled laboratory studies [27,45]). A number of acute studies with children and adults across different settings also reported an impact on consumed amounts with smaller bowls alone or alongside smaller spoons, but they all come from Cornell University [49,60,61].

Using smaller cutlery produced different results depending on the setting. For instance, smaller forks were effective in the laboratory but not in the restaurant [69], and smaller spoons were effective in adults but not in children [46,70].

Regarding glass shape and size, a mega-analysis of 8 studies reported no impact on wine sales (a surrogate for consumption) in bars, although for restaurants, a larger (350–370 mL) than standard (290–300 mL) glass induced higher wine sales [44].

Some of these studies were carried out under uncontrolled testing conditions, and the study populations ranged from children, only women, to individuals with special conditions, which may increase the heterogeneity in the results.

**Figure 5:**
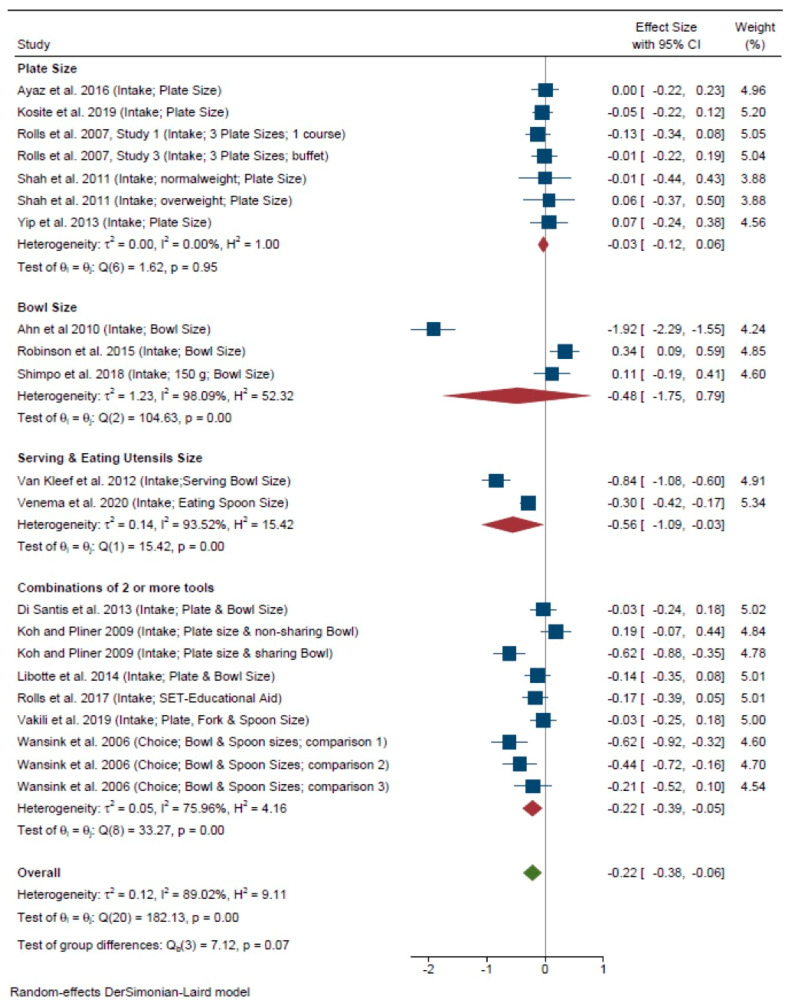
Forest plot for the analysis of all comparisons examining the effects of portion control tools on portion size intake (consumed amount) by tool type using a random-effects meta-analysis. Contributing comparisons are represented by a filled square with horizontal lines, where the area of the square depicts the contribution of the study to the full analysis, and the horizontal lines indicate the 95% CIs for each study. Studies displaced to the left of a 0 line demonstrate a finding in favor of the portion control tool to reduce food intake, whereas those to the right demonstrate a finding in favor of the portion control tool to increase consumed amounts when compared with the control condition. The diamond at the base of the plot represents the combined effect (Standardized Mean Difference) with 95% CIs. Wansink et al. 2006 Comparison 1—Small bowl combined with small spoon vs. Large bowl combined with large spoon. Wansink et al. 2006 Comparison 2—Small bowl combined with large spoon vs. Large bowl combined with large spoon. Wansink et al. 2006 Comparison 3—Large bowl combined with small spoon vs. Large bowl combined with large spoon.

Sixteen of these studies, representing 21 comparisons, were included in the meta-analysis (Figure 5), which confirmed the trend observed in the whole sample of papers. A significant overall effect of tool was detected indicating that the use of a portion control tool induces a reduction in food consumption (*d* = –0.22, 95%CI: –0.38, –0.06; I^2^ = 89.02%), but with a small effect size. A test of group differences just fell short of detecting a significant impact of tool type on food intake (χ^2^(3) = 7.12, *p* = 0.07). Close examination of the data suggests that combinations of two or more tools including reduced-size tableware (*d* = –0.22; 95% CI: –0.39, –0.05; I^2^ = 75.96%, 9 comparisons) might work in certain contexts, but this should be confirmed with further studies. Noticeably, the effect of the combined tools disappears after removing one study from Cornell University [61] (*d* = –0.13; 95% CI: –0.32; 0.06; I^2^ = 75.46%). Although a potential impact might be deduced for serving bowl and spoon size (*d* = –0.56; 95% CI: –1.09, –0.03; I^2^ = 93.52%) only two comparisons could be included in this group and data from other studies using smaller cutlery did not support this effect [46,69].

Studies comparing plates of different sizes on their own [52,54,55,56,71,72] and one long-term RCT employing a tool set (including a digital food scale, measuring cups and spoons, a placemat illustrating appropriate portions of meal components, and a portion card with common objects) [12] could not confirm an effect of either strategy on food intake.

Sensitivity analyses showed no overall impact of awareness of study purpose (test for group differences χ^2^(2) = 1.20, *p* = 0.55) or strategy, that is, employing only the tool vs. using the tool alongside another strategy (χ^2^(1) = 0.82, *p* = 0.36). Removing the study from Cornell University [61] did not change the results in either case (Supplementary Figures S2–S5). Replacing the small rice PS (150 g) in Shimpo and Akamatsu 2018 [45] with the large PS (250 g) did not change the results (Supplementary Figure S6).

### 3.6. Results of Studies Examining Weight Status

Nine studies were included in this group, all involving long-term weight management interventions (Table 6). Eight of these studies reported a positive overall impact of portion control tools on body weight status (i.e., the inclusion of the tool helped with weight loss or a change in BMI).

**Figure 6:**
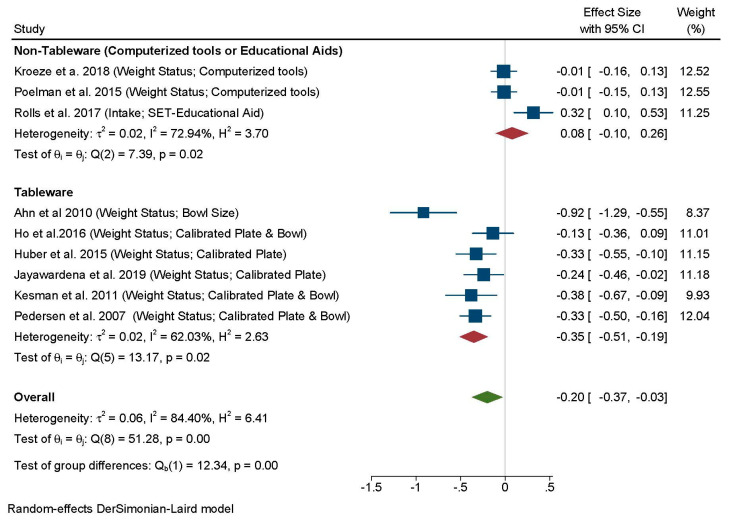
Forest plot for the analysis of all comparisons examining the effects of portion control tools on weight status (weight loss or BMI change) using random-effects meta-analysis. Contributing comparisons are represented by a filled square with horizontal lines, where the area of the square depicts the contribution of the study to the full analysis, and the horizontal lines indicate the 95% CIs for each study. Studies displaced to the left of a 0 line demonstrate a finding in favor of the portion control tool helping to reduce body weight, whereas those to the right demonstrate a finding in favor of the tool to promote a higher BMI or weight gain, when compared with the control condition. The diamond at the base of the plot represents the combined effect (Standardized Mean Difference) with 95% Cis.

All nine studies reporting weight status variables were included in the MA (Figure 6). The combined analysis showed a marginal impact on weight status (measured as body weight or BMI change), across all tools (*d* = –0.20; 95% CI: –0.37, –0.03; I^2^ = 84.40%). A test for group differences revealed a significant effect of tool type (χ^2^(1) = 12.34; *p* 0.01). In particular, four studies using calibrated plates [58,59,74,76] and one study using smaller bowls [53] were associated with reductions in body mass (BMI and body weight) with a medium effect size (*d* = –0.35; 95% CI: –0.51, –0.19; I^2^ = 62.03%, 6 comparisons including a calibrated set used in adolescents which failed to reach significance [51]). No impact was observed for computerized tools and the set of tools from one long-term RCT [12].

Given that several studies report a change in adherence to PS interventions at 3 months [12,74,76] we conducted two separate additional analyses for studies reporting outcomes at 3 months vs. at or after six months. Data from studies reporting BMI or body weight changes at 6–12 months favoured a small but significant effect of tools on weight status variables (either weight loss or BMI) (*d* = –0.15; 95% CI: –0.30, –0.00; I^2^ = 71.86%). A similar trend was visible in studies reporting BMI or body weight changes at 3 months, but it failed to reach significance (*d* = –0.19; 95% CI: –0.41, 0.02; I^2^ = 86.70%) (Supplementary Figures S7 and S8).

Sensitivity analyses examining effect of awareness of study purpose by participants could not be conducted for the weight loss studies as the interventions were overt in all cases.

### 3.7. Results of Studies Examining Meal Components

Data for the impact of tools on meal components could be extracted from 17 comparisons. No overall effect of tool was detected for vegetables (four comparisons, *d* = 0.53; 95%CI: –0.02, 1.09; I^2^ = 95.77%), carbohydrates (five comparisons, *d*= –0.35; 95%CI: –0.95, 0.26; I^2^ = 96.36%), protein (six comparisons, *d* = –0.18; 95%CI: –0.69, 0.33; I^2^ = 96.23%) and fat (two comparisons, *d* = –0.68; 95%CI: –2.14, 0.77; I^2^ = 97.78%).

### 3.8. Quality Assessment of the Studies in the Review

Information about the risk of bias (ROB) for each study is reported in the Supplementary Table S2. Thirty-eight studies out of 40 informed about the primary research question and all of them evaluated the effect of portion control tools. Most of the included studies involved a high risk of participants being aware of the study purpose because only a few of them reported the use of a cover story with many involving weight loss interventions where portion control was one of the strategies included (making it difficult for participants to remain blinded to the study purpose). On the other hand, almost all of the crossover studies were randomized and considered carry-over effects. Only seven studies declared more than 20% dropouts, and 38 reported all pre-specified outcome measures. Finally, 11 studies were judged to be at high risk of bias due to failure to conduct ITT; however, this is common in crossover studies.

Funnel plots and Egger’s tests using both random and fixed effects MA revealed no asymmetry for any studies except for those reporting weight status variables (Supplementary Table S3 and Figure S9). There was an insufficient number of studies to explore asymmetry for PS awareness studies [38]. From these results, publication bias or other sources of significant heterogeneity could not be confirmed for studies examining PS awareness, choice or intake. Visual inspection of the funnel plots for weight status studies shows there are a few more studies reporting weight loss than weight gain; however, a clear relationship between effect size and standard error does not emerge, and publication bias cannot be confirmed based on such limited number of studies [38].

## 4. Discussion

### 4.1. Main Findings

The aim of this work was to gather information on the breadth of existing portion control tools and their effectiveness in managing amounts chosen and consumed, as well as the potential for these tools to educate on portion size and induce weight loss. A wide range of utensils were identified; however, only a narrower range have been formally tested. Tools tested included in their majority, plates, bowls and spoons of smaller diameter or capacity vs. standard sizes, plus portion control (calibrated) tableware. Overall, the results showed that using portion control tools can help reduce food portion size, mainly amounts selected and consumed; however, there was a clear impact of tool type and number. We detected only a marginal impact on PS awareness, but the number of studies examining this outcome was limited. Many studies explored the effect of different portion control tools (including reduced-size tableware and technologies) in combination with other weight management strategies, making it difficult to determine the impact of the instruments on their own. Despite this, we found evidence that certain types of instruments/tools, in particular calibrated bowls and plates [53,59,76,77] and, less consistently, combinations of smaller bowls and spoons [52,61], may be useful for portion size control. The results also suggest that some technologies [65,68] have the potential to help increase portion size awareness and related behaviour, in particular, web-based educational programmes, but more studies are needed to confirm this trend. We did not find sufficient evidence demonstrating that glasses, cutlery, bowls and in particular plates of reduced diameter or capacity are effective for portion control when used in isolation, at least in adults, contradicting popular recommendations [62,82].

### 4.2. Comparison with Previous Work

Results from previous, similar reviews had been inconsistent, especially regarding the impact of reduced size tableware [18,23,24,32]. The overall evidence suggests that portion control tool effectiveness is probably mediated by the tool design and other variables that may have confounded previous analyses, including participants being aware of the study purpose, heterogeneous study designs (lab vs. free-living condition, self-served vs. fixed portion size) and type of strategy (featuring the tool alone or combined with other approaches) [5]. Specifically, two previous reviews [23,73] concluded that plate size had no reliable effect on food portion size, but a later review by Holden et al. 2016 [24] reported a significant effect on served and consumed amounts in subjects not being aware of the study purpose and when portion sizes were self-selected, as opposed to given in fixed amounts to participants. The context of eating may also act as a potential confounder, including possibility to refill the container, having second helpings [27], pouring as opposed to using a prefilled glass [44] or eating to satisfy a pre-defined goal (i.e., emptying the plate when eating at a restaurant) [69].

There was no evidence that the tool was more effective in reducing PS intake amongst the 13 studies reporting use of a covert design or cover story compared with studies with informed/aware participants These findings differ from those previously reported [24] suggesting that tool size had a stronger effect when participants were unaware that they were participating in a food study. However, in that analysis, plates, bowls and packaging were included in the same category, and therefore, results may differ. There is currently some debate as to how restrictive protocol demands should be in food eating studies [83,84]; however, it is likely that participants change their eating behaviour when they know their food intake is being monitored [85].

Specific meal components (i.e., starch, vegetable, protein) and/or eating occasion (meal vs. snack) may further modulate the impact of tools on intake [63,70,72]. For example, individuals may find it harder to reduce portion sizes when very hungry and when faced with highly palatable foods [46]. We did not detect any differential impact of tool on the portion size of specific meal components amongst this sample of studies; however, there was a high heterogeneity and a small number of studies for some analyses. We did not examine the impact of eating occasion, but this could be explored in future analyses.

#### 4.2.1. Impact of Plate and Bowl Size

In agreement with previous reviews [5,23,24], smaller plates (typically 25 cm in diameter) and bowls were not always effective in controlling portion sizes when used without other strategies, especially in adults. This may be explained by differences in study design. For example, not serving your own food [24] has been shown to reduce the impact of reduced size tableware, while palatable food may counteract the potential effects of reduced size plates. Moreover, smaller plate sizes used on vegetables dishes appear to have a low impact on intake in adults [72]. On the other hand, simultaneous strategies or contextual factors in addition to changing the bowl size may have enhanced the potential effect of the bowl in some studies [53,61].

Conflicting results might also be attributable to differences in the study population [54]. Studies involving children reported significant effects of plate and bowl size [48,49,50], while several studies assessing plate and bowl sizes in adults did not [27,45,71,73]. Young children in particular tend to respond better to internal satiety signals than older children and adults which may make them less susceptible to external cues (such as a large food container). However, a high variety and number of items (such as in commercial snack boxes for children) may counteract this effect [86]. Indeed, the portion size effect is well documented in children [46,87], and a number of the studies in children were rated as high risk of bias [48,49].

Some specific contexts and study designs may further explain the inconsistent results with plates and bowls. Bowls were effective for rice, ice cream and cereal in Korea and the USA [49,53,61] but not in Japan [45], where it is considered poor table manners to leave food on the plate. Bowls were also effective in children both at home [50] as at school [49], but not for adults eating popcorn distractedly under laboratory conditions. Moreover, in some studies, people were allowed to refill the plate or bowl [27,52,72], which could have a counteractive effect [27].

#### 4.2.2. Impact of Tools with Specific Design

The presence of printed or 3D demarcations for portion sizes of different foods, such as in calibrated plates, seemed to be effective in inducing weight loss when used as part of a wider weight management programme. Such effect may be mediated by reduced serving sizes of specific meal components and eventually less energy being consumed. We observed a medium size effect for four types of calibrated plates mostly including printed guidelines [59,74,76] or indented sectors [75], which was confirmed by self-reported data from a field study [77]. Thanks to their design, these plates may be useful in controlling portion sizes from specific meal components that tend to be overconsumed (i.e., starch, protein) while at the same time inducing larger servings of desirable foods such as fruit and vegetables, eventually acting as an educational aid for appropriate consumption [77,78]. The same impact could potentially be derived from portion control serving utensils such as calibrated serving spoons [77], although more evidence is needed in regards to these utensils. While the design of this type of tools might help learning what a suitable amount of food should look like and avoid excessive amounts being initially placed on the plate, “piling up”, second helpings and going over the depicted section or sector is still possible [78]. This underscores the importance of including an educational component in the design of any portion control tool for it to be successful, especially in the long term. Continuous use of portion control tools may be challenging in some individuals due to lifestyle demands (i.e., eating on the go, with relatives, on holiday), or they may feel that they have already learned to proportionate their meals [78]. On the other hand, calibrated tableware has been reported to be well accepted and perceived as easy to use and effective to promote behaviour change [74,76,77,78]. Despite this, its impact on learning has often been masked by additional weight management strategies applied alongside the tool [59,74,76]. In addition, analysis effects have been limited to 6 months; therefore, the longer-term impact of these tools is not known, although lack of adherence beyond 3 months is a potential cause of attenuated impact in the long-term [2]. Having a support system in the form of group meetings or more frequent contact with health professionals may improve compliance [51,59] but will increase resource and financial needs.

The actual mechanisms through which calibrated utensils may impact portion size learning and consumption are still unknown. Preliminary work in our laboratory suggests that calibrated plates may influence energy intakes via changes in behavioural and physiological parameters, at least in the short term. It is possible that calibration marks on tableware may impact on portion intake via changes in renormalization of the so-called portion distortion effect [88,89] and may also modulate the satiety response; however, further work is needed to confirm these processes.

#### 4.2.3. Impact of Glass Shape and Size

People use the relative fullness of glasses to judge volume [30]; therefore, both the shape and capacity of glasses could influence perceived volume. For example, soft drinks are drunk more quickly from an outward-sloped glass, relative to a straight-sided glass [28], possibly related to the way the lips are placed around the edge of a straight-sided glass [90]. Beyond shape, larger glasses also increase consumption [30]. Perceptions that smaller glasses contain more than larger ones (despite containing the same volume) could in theory slow drinking speed and overall consumption. However, to date, drinking speed, sip number or sip duration have not been linked with changes in PS consumption at least for wine [29]. The context of drinking seems to be a more important factor. A mega-analysis of 8 studies across 5 establishments reported no significant effect of glass size on volume of wine sold in bars in the UK while for restaurants, using 370-mL glasses increased wine sales by 7.3% relative to 300 mL glasses. However smaller (250 mL) or larger glasses (450 mL) glasses did not affect sales, maybe because most sales in restaurants are in bottles or jugs which require pouring [44]. These studies suffer from high level of confounding due to being carried out in naturalistic environments, so conclusions need to be considered with caution. Despite this, it is likely that specific shapes and reduced capacity glasses are beneficial for beverage portion size control.

#### 4.2.4. Impact of Cutlery Size

Initially, the size of the spoon or fork could modulate how many spoon- or forkfuls one takes, by influencing selected food portion sizes [61,69]. In a restaurant setting, patrons consumed more with a small fork than with a large one; however, this effect was reversed in the laboratory when offered an ad libitum portion, confirming studies on eating rate [91]. The authors suggested that the restaurant setting reversed the effect of the small utensil by inducing to eat more to comply with a pre-defined goal (i.e., becoming satiated by emptying the plate) [69]. Larger ice-cream scoops also led to increased consumption in a social event [61] and pre-school children self-served more food with a tablespoon than with a teaspoon in a controlled laboratory study [46]. However, in either case, a direct effect of the eating utensil size on intake could be confirmed, being the total amount of food served what determined intake. It is possible that confounding due to simultaneous manipulation of the portion size available in addition to cutlery size, masked the impact of the small spoon in the children study [46]. In support for this, a study in young adults identified via a separate search strategy for another review suggested that smaller cutlery may help reduce bite size and eating rate, resulting in less amount of food being consumed when exposed to an ad libitum portion [91].

#### 4.2.5. Impact of Other Instruments and Approaches

Compared with other strategies, such as using a fixed size portion (i.e., ready to eat, pre-packaged meals), the use of portion control educational guides and measuring tools did not seem to be as effective in controlling food intake and body weight in a large scale-RCT lasting 12 months [12]. These strategies, however, did help to reduce dietary energy density, which reflects that changing the type of foods rather than the amount is possibly a more realistic goal for individuals engaging in long-duration weight loss attempts. On this basis, it has been suggested that new approaches to weight management should incorporate both aspects of dietary control [92]. Calibrated plates are well suited for this purpose as their design specifically induces a reduction of the energy density of the meal [77], in particular by restricting amounts of energy dense components such as carbohydrate and protein and guiding on increasing vegetables, so their impact on energy density deserves further exploration.

Combining measuring tools with educational technologies could improve awareness and learning of recommended portion sizes which eventually may translate in improved portion size behaviour [12,65,66]. There is a high potential for the use of technologies to educate on recommended amounts and facilitate adherence to nutritional interventions, since technology will enhance availability of the tool. The impact of these strategies may fluctuate depending on the type of population to which it is directed though, and specific training may be needed for some groups [57,65,68].

### 4.3. Risk of Bias and Asymmetry

ROB analyses detected a considerable number of studies with high risk of bias however this did not translate in significant evidence of asymmetry, except for weight loss studies. As both random effects MA (which gives more weight to small studies) and fixed effects MA detected asymmetry for weight loss studies, this asymmetry is unlikely to be driven by study size. However, given the elevated heterogeneity detected (I^2^ = 84%), there may be other causes than publication bias contributing to this asymmetry. Unfortunately, we could not discern this due to the small number of studies in this category.

Despite the above, the information obtained from all the studies allows identifying relevant aspects to include in future interventions, such as standardising the type of portion size administration and separating the effect of the tool per se vs. that from other strategies applied alongside the tool. In addition, it is important to try to design studies including an ITT analysis, to be able to evaluate the impact of the dropouts in the results. Controlling for other external variables such as who is serving the food, eating with others, and presence of highly palatable foods plus previous knowledge of portion sizes is also relevant.

### 4.4. Strengths and Limitations of This Review

To our knowledge, the present work represents the most updated systematic review of existing portion control tools and related strategies to date, including a meta-analysis of the impact of these tools on several behaviours related to portion size control. Previous reviews have reported effects of portion control tools used alongside other portion control strategies and highlighted the importance of assessing covariates (i.e., participants’ awareness of the study purpose, the purpose of the tool and the type of portion size administered i.e., fixed vs. self-served). We took into account these potential variables and examined the effects of specific portion control tools on their own as well as when part of wider interventions when data were available. The results support recent findings suggesting that tools based on bi-dimensional effects (i.e., educational aids and reduced size plates) have a limited impact on portion control [23,24]; however, specific types of tools such as those giving volume specifications (i.e., calibrated tableware and serving utensils) can potentially improve portion size selection and intake, at least for certain foods. The evidence remains inconclusive as to the impact of cutlery, bowl and glass size but points out to a potential effect mediated by the context of eating.

A limitation of the present work was that not all the studies examining the impact of portion size tools could be included in the meta-analysis due to the lack of quantitative data. Although missing data and clarifications were requested from authors, only a limited range of instruments were formally tested for improving portion size awareness in particular, and most of these studies used non-tableware tools. Confounding variables were also present in many studies leading to significant heterogeneity. Cutlery size for example was frequently studied in combination with other tools such as bowls of different sizes, which could mask any specific impact of cutlery size [61]. The same applies to small plates tested alongside a sharing serving bowl, which may have lost effectiveness in reducing intake if the impact of sharing the serving bowl dominated [52]. Confounding due to manipulation of portion size alongside tool size was also a limitation [45,46]. A few studies were evaluated as having low quality due to various design issues. When missing data could not be obtained from authors, or quality was suspiciously low [93], these studies were excluded. Another limitation concerns the lack of experiential and mechanistic data for some types of tools, in particular technology-based tools and calibrated tableware, as studies testing these tools tended to focus on weight outcomes. This limits our capacity to draw conclusions on the acceptance levels and specific mechanisms by which such tools may work. Such information is essential to improve the tools design to enhance adherence and effectiveness.

Finally, most of the tools examined focused on solid or semi-solid foods (i.e., ice cream, soup, pasta), leaving aside beverages beyond wine. Large portion sizes of sugar-sweetened energy yielding beverages are a particular public health problem [94]. Despite this, the impact of portion size tools on beverages has been poorly studied. We only identified one study exploring a calibrated glass (for water, milk or juice) and a mega-analysis exploring wine glass size and shape; however, these were rated at high risk of bias [44,77].

We did not specifically look at bottle size impact, but research has shown that large bottle sizes (i.e., 750 mL vs. 500 mL) increase consumption amount and drinking speed for wine [95]. However reduction of bottle size beyond a certain threshold may be counterproductive as people may end up consuming more bottles out of convenience and portability, amongst other reasons [96].

### 4.5. Implications for Practice and Future Research

Most of the studies examined in this review focused on adults, and many were conducted in controlled conditions under laboratory settings. While some were conducted in bars, restaurants and in schools, with children and adolescents, contextual confounding was high in these studies. Despite this, studies in children suggest that portion control tools specifically designed for children could be effective for the management of overweight in this group, a pending public health target [97,98,99]. Portion management for children should also target increasing portion sizes for low energy density foods such as fruit and vegetables, which may be achieved by using larger plate sizes or larger snack packs for healthy foods [50,100,101]. The growing number of commercial portion control tools for children offers a good opportunity to address this gap [102].

Findings from this work could help improve the design of current and new instruments, in particular, tableware and serving utensils. The impact of calibrated plates in weight loss maintenance and promotion of healthy lifestyles specially should be assessed, alongside the specific mechanisms by which these tools work. This will allow designing more effective and versatile tools for the different population groups that may benefit from them (i.e., the young, the elderly, pregnant women, those with specific metabolic conditions, etc.) [103,104].

Further studies exploring the combined impact of different portion control tools on portion size and dietary energy density are warranted [2,92]. Such tools may also be used to increase dietary energy density in the elderly and combat malnutrition. Evidence supporting the benefits of regulating glass capacity could also help defining public health targets to modulate consumption of energy and alcohol [44]. Finally, further studies based on tool acceptance and tool adherence must be conducted to improve and elucidate the impact of educational tools especially in the long term.

## 5. Conclusions

Specific portion control tools, mainly calibrated tableware and some eating and serving utensils, have the potential to reduce serving sizes and consumed amounts, suggesting that their inclusion as part of weight loss trials may enhance the impact of interventions on food intake and weight loss. Strategies based solely on size manipulation may have a positive effect only when (a) they are used alongside other tools, (b) the tool provides a volume dimension (bowls, glasses), and (c) they are used in specific eating contexts and population groups. The potential impact of technology-based tools and of calibrated tableware as educational or practical tools to guide appropriate consumption is initially promising and warrants further investigation.

## Figures and Tables

**Table 1:** PICOS criteria for inclusion and exclusion of studies (based on [33]).

Criterion	Description
Population	Healthy adults and children or subjects with a controlled clinical condition not affecting their day-to-day activities.
Intervention	Any intervention in which an instrument or tool is used to control food/drink portion size irrespective of its validation status and not requiring significant professional guidance or a clinical setting for the user to be able to use it appropriately (i.e., tools providing direct feedback to the user via guidelines for appropriate consumption or by restricting the amount of food than can physically be served or consumed).
Comparison	Other tool size/design/type or control condition; no tool.
Outcome	Range of portion control tools currently available and their effect on poriton size awareness, choice and intake; weight loss, BMI change, experiential and other relevant data.
Study design	Any study design involving the application of a tool or instrument to control food portion size including 3D tools, 2D educational aids and/or technology-based tools; review papers with relevant references.

**Table 2:** Overview of the 40 studies included in the review (36 publications) with data on potential covariates (participant awareness of study purpose, type of portion size offered (self-selected vs. fixed) and presence of other strategies used alongside portion size modification). All studies were carried out in adults except when otherwise indicated under “Tool and control”. The term *calibrated* is used to describe a portion control utensil with either printed indicators or indented segments (3D). Overall impact of tool is coded as follows: Green—beneficial impact of the small or intervention tool; Orange—relative impact; No color—insufficient evidence or no impact shown. *Abbreviations:* CHO, carbohydrate; FV, fruit and vegetables; PRO, protein; PS, portion size. *Study outcome* A, Portion size awareness; C, Portion size choice; I, Portion size intake; W, Weight status.

Tool and Control	Study Outcome	Duration of Intervention	Participant´s Awareness of Study Purpose	Type of PS	Other Strategy Used Alongside Intervention	Overall Impact of the Tool	Reference
NON-TABLEWARE							
EDUCATIONAL AIDS AND MEASURING UTENSILS							
Tool set (food scales, measuring cups/spoons, placemat with image of plate depicting recommended PS, reference object PS cards). Control: Standard care	A, I, W	12 months (free-living)	Aware	Self-selected	Yes—Part of a portion control intervention (*Portion-Control Strategies Trial*)	Relative impact: NO—body weight YES—dietary energy density	Rolls et al. 2017 [12]
COMPUTERIZED TOOLS							
*ServARpreg* application for mobile phone Control: No tool	A	2 weeks	Aware	N/A (training tool)	No	Relative impact: NO—PS knowledge YES—CHO content estimation	Brown et al. 2019 [57]
*PortionSize@warenessTool,* on-line programme No control (before and after)	A, I, W	9 months	Aware	Self-selected	Yes—Part of a portion control intervention (*SMARTsize*)	YES—portion control behaviour (3 months); BMI (9 months)	Kroeze et al. 2018 [64]
*PortionSize@warenessTool,* on-line programme No control (before and after)	A	Acute study	Aware	Self-selected	Yes—Part of a portion control intervention (*PortionControl@* *HOME*)	YES	Poelman et al. 2013 [65]
*PortionSize@warenessTool),* on-line programme No control (before and after)	A, I, W	12 months (free-living)	Aware	Self-selected	Yes—Part of a portion control intervention (*PortionControl@* *HOME*)	YES (3 months)	Poelman et al. 2015 [66]
*Food Portion Tutorial* computer programme, two comparisons: (a) No training vs. training (immediately before meal); (b) No training vs. training (delayed)	A, I	Acute study	Aware	N/A (training tool)	No	NO	Riley et al. 2007 [67]
*ServAR* application for tablet vs. Verbal information on recommended PS, vs. Control: No tool	C	Acute study	Aware	Self-selected	No	YES	Rollo et al. 2017 [68]
TABLEWARE							
DIFFERENTLY SIZED TABLEWARE							
Bowls							
Small vs. standard size bowl	I, W	3 months (free-living)	Aware	Self-selected	No	YES	Ahn et al. 2010 [53]
Small vs. large bowl	C,I	Acute study (lab setting)	Unaware	Self-selected	No	NO	Robinson et al. 2015 [27]
Small vs. Large bowl Small rice portion size vs. large rice portion size	I	Acute study (lunch in a classroom)	Unaware (cover story used)	Fixed and self selected (refills)	Yes —rice portion size (small vs. large)	NO	Shimpo and Akamatsu 2018 [45]
Large vs. small cereal bowl (6–12 years old)	C	Acute study (schools)	Not reported/ insufficient information	Self-selected	No	YES	Van Ittersum and Wansink 2013 [48]
Small vs. large bowl (pre-school children)	C, I	Acute study (schools)	Unaware-not clear (with the researcher)	Self-selected	No	YES	Wansink et al. 2014 (Study 1) [49]
Large vs. small bowl (6–12 years old, deprived families)	C,I	Acute study (summer camp)	Unaware	Self-selected	No	YES	Wanskink et al. 2014 (Study 2) [49]
Cutlery and serving utensils							
Serving teaspoon vs. serving tablespoon (4–6 years old, ethnically diverse, some deprived)	C,I	Acute (lab setting)	Unaware	Self-selected	Yes—amount of entrée available	YES	Fisher et al. 2013 [46]
Small vs. large fork	I	Acute study (restaurant)	Not reported/ insufficient information	Fixed	No	NO (reverse effect detected i.e., those given small fork ate more)	Mishra et al. 2012 [69]
Small vs. large fork	I	Acute study (lab setting)	Not reported/ insufficient information	Fixed	No	YES	Mishra et al. 2012 [69]
Small vs. large spoon	C, I	Acute study	Unaware (cover story used)	Self-selected	Yes—tea served hot or cold as part of an additional research question	YES	Venema et al. 2020 [70]
Small vs. medium-size serving bowl	C, I	Acute study	Not reported/ insufficient information	Self-selected	No	YES	Van Kleef et al. 2012 [60]
Glasses							
Five glass sizes (250 mL,300, 370 mL (350 in restaurants), 450 mL and 510 mL	I	Mega-analyis of 8 acute studies (5 bars and restaurants)	Unaware	Fixed	No	Relative impact: NO—bars YES—restaurant (370 mL glass increased sales vs. 300 mL)	Pilling et al. 2020 [44]
Plates							
Small vs. medium-size vs. large plate	I	Acute study	Unaware (cover story used)	Self-selected	No	NO	Ayaz et al. 2016 [54]
Small vs. large plate	I	Acute study	Unaware (cover story used)	Self-selected	No	NO	Kosite et al. 2019 [71]
Small vs. medium-size vs. large plate	I	Acute study	Unaware (only 1 subject guessed)	Self-selected	No	NO	Rolls et al. 2007 (Study 1) [72]
Small vs. medium-size vs. large plate	I	Acute study (personal buffet)	Aware (55% of subjects guessed)	Self-selected	No	NO	Rolls et al. 2007 (Study 3) [72]
Small vs. large plate	I	Acute study	Unaware (blinded)	Self-selected	No	NO	Shah et al. 2011 [55]
Small vs. large plate	C, I	Acute study (all-you can eat Chinese buffet)	Unaware	Self-selected	No	YES	Wansink and Van Ittersum 2013 (Study 2) [62]
Small vs. large plate	C	Acute study (health conference buffet)	Unaware	Self-selected	No	YES	Wansink and Van Ittersum 2013 (Study 3) [62]
Small vs. large plate	I	Acute study (palatable buffet)	Aware	Self-selected	No	NO	Yip et al. 2013 [56]
Tool combinations							
Child-sized vs. adult tableware (plate and bowl); (4–5 years old)	C, I	~1 week	Not reported/ insufficient information	Self-selected	No	YES	DiSantis et al. 2013 [50]
Small vs. large plate with either a shared serving bowl or an individual serving bowl	C, I	Acute study	Unaware (cover story used)	Self-selected	Yes—meal eaten with a friend or stranger as part of an addition research question	YES	Koh and Pliner, 2009 (Study 4) [52]
Large vs. standard size tableware (dinner plate, bowl) with side plate	C	Acute study	Not reported/ insufficient information	Self-selected	No	Relative impact: NO—energy intake YES—larger vegetable PS	Libotte et al. 2014 [73]
Medium-size plate with standard size spoon vs. large plate with large spoon (50% more vs. standard size)	I	Acute study	Unaware	Fixed	No	NO	Rolls et al. 2007 (Study 2) [72]
Small vs. large bowl with small vs. large ice-cream scoop	C, I	Acute study (Nutritionists social event)	Unaware	Self-selected	No	YES	Wansink et al. 2006 [61]
Small vs. large tableware (plate, spoon and fork); both served with 120 mL glass	I	Acute study	Not reported	Self-selected	No	Relative impact: NO—total energy YES—rice PS reduction	Vakili et al. 2019 [63]
PORTION CONTROL/CALIBRATED TABLEWARE							
Portion control Plates							
Calibrated plate (glass with print) with tele-coaching vs. no plate and standard advice (leaflets)	I, W	6 months (free-living) (Mayo Clinic)	Aware	Self-selected	Yes—tele-coaching present	YES (3 months)	Huber et al. 2015 [74]
Calibrated plate with 5 sectors (printed) for Rice, PROT and 3 types of vegetables, vs. standard care	C, W	3 months (free-living)	Aware	Self-selected	Yes—given alongside standard care for CVD	YES (3 months)	Jayawardena et al. 2019 [58]
3D plate with indented sectors for CHO, PROT and FV vs. regular plate	C	Acute study	Aware	Self-selected	No	YES	Hughes et al. 2017 [75]
Calibrated tool combinations							
Calibrated *DietPlate* plate plus bowl vs. no tableware (both groups received nutritional counseling) (8–16 y olds)	I, W	6 months (free-living)	Aware	Self-selected	Yes—part of *FOCUS* family intervention programme	NO	Ho et al. 2016 [51]
Calibrated glass plate and bowl with print vs. standard care	I, W	6 months (free-living) (Mayo Clinic)	Aware	Self-selected	Yes —food poster and nutrition advice customized	YES (3 months)	Kesman et al. 2011 [76]
Calibrated *DietPlate* plate plus bowl and book vs. standard care (dietitian contact at start and then as needed)	I, W	6 months (free-living) (private clinic)	Aware	Self-selected	Yes—Part of a portion control intervention receiving follow-ups by dietitians and required to complete a daily log	YES	Pedersen et al. 2007 [59]
Calibrated plate, bowl and glass (*Precise Portions*) or portion control serving spoons (*Healthy Steps*) calibrated protein, carb and veggie ladles/spatula). No control (before and after)	A	2 weeks each tool (free-living)	Aware	Self-selected	No	Relative impact: NO—glass YES—plate, bowl, serving spoons	Almiron-Roig et al. 2016 [77]; 2019 [78]

**Table 3:** Studies reporting changes in portion size awareness and learning.

Authors, Country	Study Design	Tool	Population	Main Results
Almiron-Roig et al. 2016 [77]; 2019 [78] UK	Randomized crossover trial including a qualitative sub-study 4 weeks (2 weeks with each tool)	Set of calibrated crockery (plate, bowl, glass) Set of plastic serving spoons (CHO, PRO, FV)	Adults with overweight and obesity (*n* = 29)	Both sets of tools were well accepted and perceived to be effective, especially to increase PS of vegetables and reduce PS of CHO. Both tools considered to be practical to help learn appropriate PS
Brown et al. 2019 [57] Australia	Baseline Survey (1 day) Parallel intervention (4 weeks)	*ServARpreg* app (mobile phone-based nutrition educational tool to assess knowledge of carbohydrates and standard serving sizes of pregnant women) vs. control group (did not use the app)	Pregnant women *n* = 186 Survey *n* = 97 Intervention (of which *n* = 36 App; *n* = 61 Control)	*ServARpreg* app improved CHO quantification knowledge (36 food items) but did not improve standard portion size knowledge (11 food items and recipes)
Kroeze et al. 2018 [64] Netherlands	Observational Study 9 months	Web based *PortionSize@warenessTool* (educational on-line program consisting on a digital dish-up for knowledge and awareness of portion size) as part of a combined educational intervention consisting of two phases (3 and 9 months, details in Supplementary Table S1) *(*SMARTsize)	Adults with overweight and obesity (*n* = 225)	Intervention improved self-reported strategies to control portion size after 3 months (i.e., prepare low-calorie dishes, intention to consume smaller portions and the use of portion control strategies). Individual counseling had no impact on hypothesized outcomes
Poelman et al. 2013 [65] Netherlands	Randomized controlled trial including online questionnaire, assessed at baseline and 1 week after	Web based *PortionSize@warenessTool* (educational on-line program consisting on a digital dish-up for portion-size knowledge and awareness) as part of a combined educational intervention (PortionControl@HOME)	Adults with overweight and obesity *n* = 167 Intervention *n* = 143 Control	Intervention enhanced the awareness of reference PS and of overeating triggers for larger portions
Poelman et al. 2015 [66] Netherlands	Parallel randomized controlled trial 12 months	Web based *PortionSize@warenessTool* (educational on-line program consisting on a digital dish-up for portion-size knowledge and awareness) as part of a combined educational intervention (PortionControl@HOME)	Adults with overweight and obesity (*n* = 278)	Intervention led to improvements on portion size awareness at 3, 6 and 12 months that induced a small reduction in BMI at 3 months of intervention. These differences were not maintained at 6 and 12 months
Riley et al. 2007 [67] USA	Parallel randomized controlled trial (12 months) Crossover trial (1 day)	CFPT —*Computerized Food Portion Tutorial* (Computer-based program providing multimedia training and feedback regarding food portions of common food items)	Adults with overweight and obesity (*n* = 76)	CFPT program modulated and improved the variation/error between the estimated and weighed portions however it failed to improve accuracy in the estimation
Rolls et al. 2017 [12] USA	Three-arm randomized controlled trial 12 months	1st arm: Tool set and educational guidelines (Digital food scale; measuring cups and spoons; placemat illustrating appropriate proportions of meal components; Portion size card with common objects) as part of the Portion-Control Strategies Trial. 2nd arm: Preportioned food group 3rd arm: Standard advice (control)	Adults with overweight and obesity (*n* = 186; *n* = 62 per arm)	The tool set and guidelines helped reduce energy density of the diet however there were no significant differences in body weight compared with the Standard advice (control group) or the pre-portioned group (alternative intervention which was the most effective at 3 months).

Abbreviations: CHO, carbohydrate; FV, fruit and vegetables; PRO, protein; PS portion size. The term *calibrated* is used to describe a portion control utensil with either printed indicators or segments separated with raised edges (3D). For full details please see Supplementary Table S1.

**Table 4:** Studies reporting changes in portion size choice (self-selected portion size).

Authors, Country	Study Design	Tool	Population	Main Results
Almiron-Roig et al. 2016 [77]; 2019 [78] UK	Randomized crossover trial including a qualitative sub-study 4 weeks (2 weeks with each tool)	Set of calibrated crockery (plate, bowl, glass) Set of plastic serving spoons (CHO, PRO, FV)	Adults with overweight and obesity (*n* = 29)	Both tools increased PSs of vegetables and helped decrease PSs of chips and potatoes (self-reported data)
DiSantis et al. 2013 [50] USA	Randomized crossover trial 8 days (school lunch)	Dishware sizes: Child-size plate (7,3”) and bowl (8 oz) Adult-size plate (10.3”) and bowl (16 oz)	(5–6 y old children) (*n* = 42)	Chid-size dishware reduced self-served PSs when compared to adult-size dishware. Food liking and meal format (unit entrée) enhanced this effect
Fisher et al. 2013 [46] USA	2 × 2 Randomized crossover trial	Serving spoon sizes: tablespoon and teaspoon Amount of entrée available: 275g and 550g	4–6 y old children (*n* = 60)	Teaspoons reduced entrée serving size by 11.5% vs. using tablespoons. Exposure to larger PS of entrée increased serving size by 40%.
Hughes et al. 2017 [75] USA	Two randomized crossover trials 1 day	3D plate (21 cm) with indicators for CHO, PRO and FV. Regular plate (30 cm) With USDA guidelines (Study 1) or household measure guidelines (Study 2) (instructions required for correct usage)	Healthy adults *n* = 70 Study 1 *n* = 40 Study 2	Calibrated plate reduced self-selected PSs of all foods. Vegetables serving sizes remained below the recommended portion sizes on both dishes
Koh and Pliner, 2009 (Study 4) [52] Canada	Mixed-methods randomized controlled trial (crossover and parallel) 1 day	Large Plate (23.5 cm) Small Plate (18.2 cm) Serving bowl, non-shared Serving bowl, shared	Women, with and without overweight (*n* = 57)	The small plate (but not the large) induced participants to self-serve less in the sharing condition vs. the non-sharing condition. Eating with friends led to self-serving more food than eating with strangers (effect of acquaintance)
Kroeze et al. 2018 [64] Netherlands	Observational Study 9 months	Web based *PortionSize@warenessTool* (educational on-line program consisting on a digital dish-up for poerion size knowledge and awareness) as part of a combined educational intervention consisting of two phases (3 and 9 months (*n*=66, 3 months; *n*=159, 9 months; see Table S1) (*SMARTsize*)]	Adults with overweight and obesity (*n* = 225)	Intervention improved self-reported strategies to control food portion size after 3 months (i.e., prepare low-calorie dishes, intention to consume smaller portions and the use of portion control strategies). Individual counseling had no impact on outcomes
Libotte et al. 2014 [73] Switzerland	Parallel randomized controlled trial (fake buffet) 1 day	Dishware sizes: Standard Plate (27 cm), bowl (14 cm), plate (16 cm) Large plate (32 cm), bowl (14 cm), plate (16 cm)	Adults, normal weight (*n* = 83)	Plate size did not have an effect on self-served total energy of the meal. Large plate promoted larger serving sizes for vegetables
Robinson et al. 2016 [27] U.K.	Parallel randomized controlled trial 1 day	Large bowl (18 cm) Small bowl (16 cm)	Adults with normal weight and overweight *n* = 31 Small bowl *n* = 30 Large bowl	The small bowl induced participants to self-serve more popcorn (4 times) vs. the large bowl (3.5 times)
Rollo et al. 2017 [68] Australia	Three-arm randomized controlled trial	*ServAR technological tool* (Augmented reality educational tool to guide the serving of food for portion control on tablet). Control group (no intervention)	Adults with normal weight and overweight (*n* = 90)	*ServAR* tool was well accepted and found easy to use. Moreover, it improved accuracy and consistency of PS estimates compared to the information and control group (actual data on serving sizes not reported)
Van Kleef et al. 2012 [60] USA	Parallel randomized trial 1 day	Large Serving Bowl (6.9 L)s Medium Serving Bowl (3.8 L)	Normal weight undergraduate students Large Bowl (*n* = 37) Medium Bowl (*n* = 30)	Large-size serving bowls promoted to self-serve 77% more pasta vs. the medium-size bowls (reduction of 44% with the small bowl)
Van Ittersum and Wansink 2013 [48] USA	Randomized crossover trial 4 days (school)	Large Bowl (16 oz) Small Bowl (12 oz)	6–12 y olds classed as extroverted or intoverted (*n* = 18)	Small bowl reduced cereal self-served PSs by 44%, especially for extroverted children
Wansink et al. 2006 [61] USA	Parallel semi-randomized trial 1 day (professional social event)	Small Bowl (17 oz) with small (2 oz) or large (3 oz) ice-cream scoop Large Bowl (34 oz) with small (2 oz) or large (3 oz) ice-cream scoop	Adults (Nutrition experts) (*n* = 85)	Small bowl reduced self-served ice cream PSs by 24%. The small ice-cream scoop reduced (a) the amount of self-served ice cream by 12% regardless of bowl size (effect most notable with the small bowl); and (b) the amount loaded onto each scoop (2.2 vs. 3 oz). Although the small spoon increased the number of tablespoons, this was not enough to increase consumption
Wansink and Van Ittersum 2013 (Study 2) [62] USA	Observational Study 1 day (Chinese buffet restaurant)	Large Plate (29 cm) Small Plate (25 cm)	Adults with overweight (*n* = 43)	Eating with a small plate reduced total energy intake by 34%
Wansink and Van Ittersum 2013 (Study 3) [62] USA	Parallel trial 1 day (conference buffet on changing health behavior)	Large Plate (29 cm) Small Plate (25 cm)	Adults (*n* = 209)	Eating with the small-size plate reduced self-serving food volume (number of trays served at group level). The large plate increased the amount of meat and fish served as well as vegetables and salad
Wansink et al. 2014 (Study 1) [49]	Parallel randomized controlled trial 1 day (schools)	Small Bowl (8 oz) Large Bowl (16 oz)	Pre-school aged children with obesity (*n* = 69)	Children requested less cereal (served by adults) with small bowl (reduction of 47%).
Wanskink et al. 2014 (Study 2) [49]	Randomized crossover trial 2 days (summer camp)	Small Bowl (8 oz) Large Bowl (16 oz)	6–12 y old children (Low-income families) (*n* = 18)	The small bowl reduced the amount self-selected (served by adults) by 41% compared to the large bowl.

Abbreviations: CHO, carbohydrate; FV, fruit and vegetables; PRO, protein; PS portion size. The term *calibrated* is used to describe a portion control utensil with either printed indicators or segments separated with raised edges (3D). The term *serving size* is used as a proxy for self-selected portion size, as stated in the original publication. For full details please see Supplementary Table S1.

**Table 5:** Results of studies reporting changes in portion size intake (in consumed amounts or energy).

Authors, Country	Study Design	Tool	Population	Main Results
Ahn et al. 2010 [53] Korea	Randomized crossover trial 3 months (at home)	Regular Bowl (380 mL) Small Bowl (200 mL)	Adult women with type 2 diabetes (with and without overweight/obesity) (*n* = 42)	The small bowl reduced total energy consumed and carbohydrate intake (in addition to body weight and blood glucose levels)
Ayaz et al. 2016 [54] Turkey	Randomized crossover trial 3 days (buffet)	Large Plate (28 cm) Medium Plate (23 cm) Small Plate (19 cm)	Normal weight Women (*n* = 37)	No effect of plate size on energy intake or on specific macronutrient intake
DiSantis et al. 2013 [50] USA	Randomized crossover trial 8 days (school lunch)	Dishware sizes: Child-size Plate (7,3”) and bowl (8 oz) Adult-size Plate (10.3”) and bowl (16 oz)	4–5 y old children (*n* = 42)	Child-size dishware reduced total energy consumed when compared to adult-size dishware. Adult-size dishware induced an increase of 0.43 kcal consumed for each additional kcal served
Fisher et al. 2013 [46] USA	2 × 2 Randomized crossover trial	Serving spoon sizes: tablespoon and teaspoon Amount of entrée available: 275g and 550g	4–6 y old children (*n* = 60)	No effect of spoon size was reported on food intake. Larger served PS tended to induce higher consumption.
Koh and Pliner, 2009 (Study 4) [52] Canada	Mixed-methods randomized controlled trial (crossover and parallel) 1 day	Large Plate (23.5 cm) Small Plate (18.2 cm) Serving bowl, non-shared Serving bowl, shared	Women, with and without overweight (*n* = 57)	The small plate (but not the large) induced participants to self-serve and eat less in the sharing condition only. Eating with friends led to self-serving more food than eating with strangers (effect of acquaintance).
Kosite et al. 2019 [71] UK	Parallel randomized controlled trial 1 day	Large Plate (29 cm) Small Plate (23 cm)	Adults with overweight and obesity (*n* = 67 per group)	No effect of plate size on total energy intake or eating parameters i.e. eating rate, bite size). Participants using the large plate left more food (average 8.6 g (95% CI [1.1, 16.0]) on the plate.
Mishra et al. 2012 [69] USA	Parallel trials (field study and controlled lab setting) 1 d	Small fork (20% less capacity than regular fork) Large fork (20% more capacity)	Adults (sample not reported) Lab study (*n* = 81)	Smaller fork increased food consumption compared to the large size fork when used in restaurant setting. Opposite pattern was found in the lab where pasta consumption was decreased with the small fork.
Pilling et al. 2020 [44] UK	Mega-analysis of 8 studies across 5 establishments	Wine glasses size (bars) Size 4 (450 mL) Size 3 (370 mL) Size 2 (310 mL) Size 1 (250 mL) Wine glasses size (restaurants) Size 4 (510 mL) Size 3 (450 mL) Size 2 (370 mL) Size 1 (250 mL)	Adults	No impact of glass size on wine sales seen in bars. For restaurants, only the 370 mL glass (and close volumes, i.e., 350 mL) increased wine sales when compared with the standard size glass (300 mL)
Robinson et al. 2016 [27] UK	Parallel randomized controlled trial 1 day	Large bowl (18 cm) Small bowl (16 cm)	Adults with normal weight and overweight *n* = 31 Small bowl *n* = 30 Large bowl	No effect size of bowl size was reported on food consumption
Rolls et al. 2007 (Study 1) [72] USA	Randomized crossover trial 3 days	Large Plate (26 cm) Medium Plate (22 cm) Small Plate (17 cm) 1 course, self-selected PS	Adults with overweight and obesity (*n* = 45)	No effect of plate size on meal energy intake
Rolls et al. 2007 (Study 2) [72]	Randomized crossover trial 2 days	Large Plate (26 cm cm) and soup spoon (50% larger than the standard) Medium Plate (22 cm) and standard spoon 1 course, fixed PS	Adults with overweight and obesity (*n* = 30)	No effect of plate or spoon size on meal energy intake
Rolls et al. 2007 (Study 3) [72]	Randomized crossover trial 3 days	Large Plate (26 cm) Medium Plate (22 cm) Small Plate (17 cm) Buffet, self-selected PS	Adults with overweight and obesity (*n* = 44)	No effect of plate size on meal energy intake
Rolls et al. 2017 [12] USA	Three-arm randomized controlled trial 12 months	1st arm: Tool set and educational guidelines (Digital food scale; measuring cups and spoons; placemat illustrating appropriate proportions of meal components; portion size card with common objects) as part of the Portion-Control Strategies Trial. 2nd arm: Preportioned food group 3rd arm: Standard advice (control)	Adults with overweight and obesity (*n* = 186; 62 per arm)	Only pre-portioned food group reduced food intake (by 11%). All groups showed a significant decrease on food energy density, but no difference was detected across groups after 3 months.
Shah et al. 2011 [55] USA	Parallel randomized controlled trial 2 days	Large Plate (27 cm) Small Plate (22 cm)	Women with and without overweight and obesity (*n* = 20)	Plate size did not impact on the amount of energy consumed, the taste of the menu, satiety or subjective appetite, regardless of body weight
Shimpo and Akamatsu 2018 [45] Japan	Randomized crossover trial 4 days	Bowl Size Large Bowl (13.5 cm) Small Bowl (11.5 cm) Rice Portion Size Small (150 g) Large (250 g)	Men with normal weight and overweight (*n* = 21)	Rice portion size had a significant effect on intake whereas bowl size did not affect rice consumption. Exposure to large portion size promoted rice consumption
Vakili et al. 2019 [63] Iran	Parallel randomized controlled trial 2 days	Ceramic/glass tableware: Large plate, spoon and fork (25 cm; 15 mL); glass 120 mL Small plate, spoon and fork (19.5 cm; 5 mL); glass 120 ml	Clerical staff of the university with overweight and obesity (n = 40)	The small tableware reduced rice consumption, but no effect was found on total energy intake
Van Kleef et al. 2012 [60] USA	Parallel randomized trial 1 day	Medium serving bowl (3.8 L) Large serving bowl (6.9 L)	Normal weight undergraduate students Large Bowl (*n* = 37) Medium Bowl (*n* = 30)	Large-size serving bowls led to consume 71% more pasta vs. medium bowls (reduction of 42% with medium bowls)
Venema et al. 2020 [70] Netherlands	Mixed-methods randomized trial (Crossover for spoon size and parallel for habit context condition) 2 days	Large spoon (5ml) Small spoon (2.5 mL) Both spoon size with either habit context disruption (cold tea) or habit context preservation (hot tea).	Adults (*n* = 123)	Participants consumed less sugar added to the tea (27%) when they used the small-size spoon. This effect was attenuated in people with a stronger habit of adding a fixed amount of sugar to tea
Wansink and Van Ittersum 2013 (Study 2) [62] USA	Observational Study 1 day (Chinese restaurant-buffet)	Large Plate (29 cm) Small Plate (25 cm)	Adults with overweight (*n* = 43)	Eating with a small plate reduced total energy intake by 31% and leftovers by 38%. The effect could be partly influenced by baseline hunger levels
Wansink et al. 2006 [61] USA	Parallel semi-randomized trial 1 day (professional celebration)	Small Bowl (17 oz) with small (2 oz) or large (3 oz) ice-cream scoop. Large Bowl (34 oz) with small (2 oz) or large (3 oz) ice-cream scoop.	Adults (Nutrition Experts) (*n* = 85)	Small bowl reduced self-served ice cream PSs by 24%. The small ice-cream scoop reduced (a) the amount of self-served ice cream by 12% regardless of bowl size (effect most notable with the small bowl); and (b) the amount loaded onto each scoop (2.2 vs. 3 oz). Although the small spoon increased the number of tablespoons, it did not increase consumption
Wansink et al. 2014 (Study 1) [49] USA	Parallel randomized controlled trial 1 day (schools)	Small Bowl (8 oz) Large Bowl (16 oz)	Pre-school age children with obesity (*n* = 69)	Children requested and ate less cereal with small bowl (served by adults) compared to large bowl (reduction of 47%)
Wanskink et al. 2014 (Estudio 2) [49]	Randomized crossover trial 2 days (summer camp)	Small Bowl (8 oz) Large Bowl (16 oz)	6–12 y old children (Low-income families) (*n* = 18)	The small bowl reduced the amount self-selected and consumed (served by adults) by 41% compared to the large bowl
Yip et al. 2013 [56] New Zealand	Randomized crossover trial 2 days	Large Plate (27 cm) Small Plate (20 cm)	Women with overweight and obesity (*n* = 20)	Plate size did not impact energy or macronutrient consumption at mealtime (buffet with attractive foods).

Abbreviations: CHO, carbohydrate; FV, fruit and vegetables; PRO, protein; PS, portion size. The term *calibrated* is used to describe a portion control utensil with either printed indicators or segments separated with raised edges (3D). The term *serving size* is used as a proxy for self-selected portion size, as stated in the original publication.

**Table 6:** Results of studies reporting changes in body weight (change in kg or BMI).

Authors, Country	Study Design	Tool	Population	Main Results
Ahn et al. 2010 [53] Korea	Randomized crossover trial 3 months (at home)	Regular Bowl (380 mL) Small Bowl (200 mL)	Adult women with type 2 diabetes (with and without overweight/obesity) (*n* = 42)	Both groups reported significant reduction on body weight after 12 weeks. No significant differences were found among groups
Ho et al. 2016 [51] Canada	Parallel randomized controlled trial 6 months (families)	Calibrated tableware (The *Diet Plate-*plate and bowl) + nutritional counseling (FOCUS intervention) Control: only counseling	8–16 y old children with overweight *n* = 51 Intervention *n* = 48 Control	Both groups reported weight loss at 6 months, but no effect of tableware was found on BMI z-score
Huber et al. 2015 [74] USA	Parallel randomized controlled trial 6 months (Mayo Clinic)	Calibrated tableware (transparent glass with guidelines and text) and tele-coaching Usual care	Adults with obesity *n* = 45 Intervention *n* = 45 Control	The combined use of tele-coaching and calibrated tableware reduced women’s body weight and BMI at 3 months. The effect did not persist at 6 months. Only a reduction in the hip-waist ratio was detected in men at 3 months
Jayawardena et al. 2019 [58] Sri Lanka	Parallel Randomized controlled trial 3 months	Calibrated plate (printed indicators) (10.5”) divided into 5 segments (rice, PRO,3 types of vegetables) Standard Care (no plate)	Adults with acute coronary syndrome *n* = 40 Intervention *n* = 39 Control	Calibrated plate reduced BMI at 3 months of intervention compared with the control condition, especially in patients with overweight and obesity
Kesman et al. 2011 [76] USA	Parallel randomized controlled trial 6 months (Mayo Clinic)	Calibrated tableware (transparent glass with guidelines text) and dietary counseling Usual care	Adults with obesity *n* = 33 Intervention *n* = 32 Control	Intervention including calibrated tableware induced greater post-treatment weight loss at 3 months, compared with conventional treatment. Effects did not persist at 6 months
Kroeze et al. 2018 [64] Netherlands	Observational Study 9 months	Web based *PortionSize@warenessTool* (educational on-line program consisting on a digital dish-up for poerion size knowledge and awareness) as part of a combined educational intervention consisting of two phases (3 and 9 months [*n*=66, 3 months; *n*=159, 9 months; see Table S1 (SMARTsize)]	Adults with overweight and obesity (*n* = 225)	Intervention improved self-reported strategies to control food portion size after 3 months resulting in 6.6% weight loss. Individual counseling had no impact on outcomes
Pedersen et al. 2007 [59] Canada	Parallel randomized controlled trial 6 months (Private clinic)	Calibrated tableware (plate and bowl) with demarcations and illustrations (*The Diet Plate*); dietary assessment and book. Usual care	Adults with obesity and type 2 diabetes *n* = 65 Intervention *n* = 65 Control	Calibrated tableware improved cholesterol and blood pressure levels, reduced the use of hypoglycemic medication and facilitated weight loss (5% of body weight or more—significant only in patients using insulin)
Poelman et al. 2015 [66] Netherlands	Parallel randomized controlled trial 12 months	Web based *PortionSize@warenessTool* (educational on-line program consisting on a digital dish-up for portion size knowledge and awareness) as part of a combined educational intervention (PortionControl@HOME)	Adults with overweight and obesity *n* = 139 Intervention *n* = 139 Control	The intervention showed improvements on portion size awareness at 3, 6 and 12 months that induced a small reduction in BMI at 3 months of intervention. These differences were not maintained at 6 and 12 months
Rolls et al. 2017 [12] USA	Three-arm randomized controlled trial 12 months	1st arm: Tool set and educational guidelines (digital food scale; measuring cups and spoons; placemat illustrating appropriate proportions of meal components; portion size card with common objects) as part of the Portion-Control Strategies Trial. 2nd arm: Preportioned food group 3rd arm: Standard advice (control)	Adults with overweight and obesity (*n* = 186; 62 per arm)	Using the tool set and the educational guides did not impact on weight status more than receiving advice (control group) or pre-portioned foods (most effective intervention at 3 months). However, all three interventions helped decrease dietary energy density and cardio-metabolic risk factors.

The term *calibrated* is used to describe a portion control utensil with either printed indicators or segments separated with raised edges (3D).

## Data Availability

Not applicable.

## Supplementary Materials

The following are available online at https://www.mdpi.com/article/10.3390/nu13061978/s1, Search strategy; computation of SMD/effect size; Table S1: Details of papers reporting changes in portion size awareness, selection and consumption with the use of portion control utensils; Tables S2: ROB- Risk of bias analysis (adapted from Cochrane guidelines); Tables S3: Egger ´s test for funnel plot asymmetry; Figure S1: Sensitivity analysis for studies examining the impact of portion control tools on portion size choice excluding data from Fisher et al. (2013); Figure S2: Impact of portion control tools on portion size intake by study purpose awareness. Figure S3: Sensitivity analysis for studies examining the impact of portion control tools on portion size intake by purpose awareness excluding data from Wansink et al. (2006); Figure S4: Impact of portion control tools on portion size intake by strategy; Figure S5: Sensitivity analysis for studies examining the impact of portion control tools on portion size intake by strategy excluding data from Wansink et al. (2006); Figure S6: Sensitivity analysis for studies examining the impact of portion control tools on portion size intake by tool type; Figure S7: Impact of portion control tools on weight status by study duration (3 months outcome); Figure S8: Impact of portion control tools on weight status by study duration (6 months outcome); Figure S9: Funnel plots for studies exploring weight status using random-effects (left) and fixed- effects (right) meta-analysis).
