# Supplementary material for: Impact of Portion Control Tools on Portion Size Awareness, Choice and Intake: Systematic Review and Meta-Analysis
Source: Nutrients. 2021 Jun 9;13(6):1978. doi: 10.3390/nu13061978 (PMC8229078; doi:10.3390/nu13061978)

1. Search Stragegy

Search equations used:

Tableware AND portion control AND portion size;

Tools AND portion control AND portion size;

Calibrated AND tools AND portion control AND portion size;

Calibrated AND serving size AND tools;

Calibrated AND tableware AND portion control;

Calibrated AND tools AND portion control;

Intervention AND portion control AND portion size;

Intervention AND tools AND portion control;

Software AND portion control AND portion size.

"Dishware" was used separately following application of all the above equations.

2. Computation of SMD / Effect size

The SMD is a measure of effect size that accounts for variability from the use of different outcome scales by estimating the difference between two experimental conditions for an outcome variable (e.g. food intake) and dividing that difference by the SD of the outcome variable for the two experimental conditions

For studies with a between-subjects design we used:

$$\boldsymbol{SMD} = \frac{\boldsymbol{Mean1} - \boldsymbol{Mean2}}{\boldsymbol{SDdiff}} \qquad \text{Equation 1}$$

Where Mean 1 and Mean 2 are the mean of the comparisons of the smaller or control tool vs. control condition, respectively. SDdiff is the SD of the difference between the means.

For studies with a within-subjects design (cross-over) we applied an adjustment for correlation between conditions:

$$\boldsymbol{SMD} = \left(\frac{\boldsymbol{Mean1} - \boldsymbol{Mean2}}{\boldsymbol{SDdiff}}\right) * \left(\sqrt{\boldsymbol{2\ x\ (1 - r)}}\right) \qquad \text{Equation 2}$$

Where Mean 1 and Mean 2 are the mean of the smaller or control tool vs. control condition, respectively. SDdiff is the SD of the difference between the means and Corr is an estimated correlation factor of 0.8, based on data compiled in Robinson et al. (2014)[1] showing that correlation in this type of cross-over studies ranges between 0.76 and 0.93.

For between-subjects studies, we computed the standardized deviation difference as:

$$\boldsymbol{SDdiff} = \sqrt{(\boldsymbol{SD1})^2 + (\mathbf{SD2})^2} \qquad \text{Equation 3}$$

As in the SDM formula, an adjustment was applied for the studies with a within-subjects design (cross-over):

$$\boldsymbol{SDdiff} = \sqrt{(\boldsymbol{SD1})^2 + (\mathbf{SD2})^2} - (\mathbf{2} * \mathbf{Corr} * \mathbf{SD1} * \mathbf{SD2}) \qquad \text{Equation 4}$$

We computed the standard error of the standard deviation difference as:

$$\boldsymbol{SE} = \frac{\boldsymbol{SDdiff}}{\sqrt{\boldsymbol{n1+n2}}} \qquad \text{Equation 5}$$

The final correction factor ($\sqrt{\mathbf{2}(\mathbf{1}-\boldsymbol{r})}$, assumes constant variance across repeated measures, and was applied as recommended by Lakens (2013)[2], using r=0.8 (Robinson et al., 2014)[1]). SMDs are reported as Cohen´s *d* in the text.

[1] Robinson E, Nolan S, Tudur-Smith C, Boyland EJ, Harrold JA, Hardman CA, et al. Will smaller plates lead to smaller waists? A systematic review and meta-analysis of the effect that experimental manipulation of dishware size has on energy consumption. Obes Rev. 2014;15(10):812–21.

[2] Lakens D. Calculating and reporting effect sizes to facilitate cumulative science: A practical primer for t-tests and ANOVAs. Front Psychol. 2013 Nov 26;4:863. doi: 10.3389/fpsyg.2013.00863.

3. Supplementary Tables

Table S1. Details of papers reporting changes in portion size awareness, selection and consumption with the use of portion control utensils. Abbreviations: *Type of study:* RCT-BS- randomized control trial- between subjects**;** RCT-WS- randomized control trial- within subjects**;** CS- Cross-sectional study**.** *Sample***:** NW- participant with normal weight OW- with overweight**;** O- with obesity. For publication details see Table 2 and Reference list in main manuscript.

Authors and country	Study design and duration	Tool type and description	Study Aim	Sample description	Main Results
PS Awareness					
Almiron-Roig et al. 2016: 2019<br><br>*UK*	Randomized crossover trial including a qualitative sub-study<br>4 weeks (2 weeks with each tool)	***<u>SET</u>***<br>Set of plastic serving utensils; and a set of crockery tableware	Acceptability and pattern of use of two portion control sets (serving utensils and tableware) by individuals with obesity	Adult-Both sexes<br>25-86 y (49 ±13)<br>OW, O<br>Healthy<br>n= 29	Both tools were perceived to be effectiveness, easy to use, well accepted (all comparisons p>0.05), and as practical strategies for portion size knowledge.<br>Using both tools increased vegetable portion size (raw and cooked) and helped reduce self-selected carbohydrates (potatoes and chips).
Brown et al. 2019<br><br>*Australia*	Baseline Survey (1 day)<br><br>Parallel intervention (4 weeks)	***<u>Non-Tw- Comp. Tool</u>***<br>ServARpreg app (mobile phone-based nutrition educational tool to assess knowledge of carbohydrates and standard serve sizes of pregnant women) vs. control group (did not use the app)	Baseline surve: assess knowledge of carbohydrates and standard serve sizes of pregnant women<br><br>Intervention: Evaluate the usability and effectiveness of ServARpreg app in improving carbohydrate and standard serve size knowledge in pregnant women.	Adult-Pregnant Women<br>30.9 ± 4.7 y<br>n= 186 (survey)<br>n= 97 (Intervention)<br>• App (n= 36)<br>• Control (n= 61)	ServARpreg app significantly improved carbohydrates quantification knowledge (p< 0.001) across 36 food items. This difference was not detected on standard serve sizes of any of 11 food items or recipes included in the study (p>0.05).<br>Most of the participants reported an increase on portion size awareness.<br>Women found ServAR as a simple, easy to use, and helpful survey for portion size awareness.
Kroeze et al. 2018	Observational Study<br>9 months	***<u>Non-Tw- Comp. Tools</u>***<br>Web based<br>*PortionSize@warenessTool*	Enhance an adequate portion control behaviour to decrease	Adult-Both sexes<br>(41.8 ± 9.9) y<br>OW, O-I,II	Self-reported strategies to control food portion size significantly improved after 3 months of intervention ($p < 0.001$) (e.g. prepare low- calorie dishes, intention to consume smaller portions and

Netherlands		(educational on-line program consisting on a digital dish-up for poerion size knowledge and awareness) as part of a combined educational intervention consisting of two phases (3 and 9 months) [3] (**SMARTsize**)	energy intake and body weight.	Healthy<br>n=225	the use of portion control strategies). In addition, an improvement of self-reported BMI was reported from baseline to 9 months (*p*< 0.001).<br>Individual counselling had no impact on outcomes.<br>Finally, almost 90% of the participants reported they used the website and were satisfied with the intervention (grade between 7.2 and 8.o on a 10-point scale).
Poelman et al. 2013<br><br>*Netherlands*	Randomized controlled trial including online questionnaire, assessed at baseline and 1 week after.	***Non-Tw- Comp. Tools***<br>Web based PortionSize@warenessTool (educational on-line program consisting on a digital dish-up for portion-size knowledge and awareness) as part of a combined educational intervention (PortionControl@HOME)	Evaluate the effect of using an interactive web-based multi-component educational tool on awareness of reference portion sizes	Adult-Both sexes<br>45.65± 9.20<br>OW, O<br>Healthy<br>n=310 (intervention n=167; control n=143	The intervention enhanced the awareness of portion-size reference (p<0.001) and of overeating triggers for larger portions (p<0.001).<br><br>Awareness of references serving sizes posttest score (range 0-12) (Mean ± SD):<br>• Intervention group: 6.31 ± 1.92<br>• Control group: 5.43 ± 2.02<br><br>Awareness of overeating triggers for larger portions posttest score(range 0-10) (Mean ± SD)::<br>• Intervention group: 4.89 ± 2.02<br>• Control group: 3.66 ± 2.33<br><br>Participants completed 40% of the modules that make up the program. The program was reported as more user-friendly when the BMI increased and the age decreased.
Poelman et al. 2015<br><br>*Netherlands*	Parallel randomized controlled trial 12 months	***Non-Tw- Comp. Tools***<br>Web based *PortionSize@warenessTool* (educational on-line program consisting on a digital dish-up for portion size knowledge and awareness) as part of a combined educational intervention (**PortionControl@HOME**)<br>Control group: wait-list control group	Evaluate the effect of using an interactive web-based multi-component educational tool on body mass index and control behaviour.	Adult-Both sexes<br>18-60 y (45.65± 9.20)<br>OW, O<br>Healthy<br>n=278<br>• Intervention (n=139)<br>• Control (n=139)	Mean BMI decreased in both groups between baseline and 3 months. These differences were not maintained at 6 and 12 months BMI remained below baseline values.<br>The intervention showed an impact on portion control behaviour and mediation effect.<br>Portion control behaviour significantly mediated the intervention effect on BMI (p<0.001). The mean estimated differences in BMI were 0.33 (95%CI=0.23 to 0.43), 0.30 (95 %CI=0.19 to 0.40) and 0.35 (95 %CI=0.24 to 0.45) at 3, 6, and 12 months, respectively, between the intervention and the control group.<br>Almost all participants (90%) logged on to the website. Different adherence levels were reported for each intervention items

[3] n=66 (online program, cooking lessons and other strategies during 3 months); n=159 (9 months, the same as the 3-month group but with individual counseling).

					(cooking classes, read educational book, filled-out the Home-Screener completely, etc.).
Riley et al. 2007<br><br>*USA*	Parallel randomized controlled trial (12 months)<br>Cross-over trial (1 day)	***<u>Non-Tw- Comp. Tools</u>***<br>The Computerized Food Portion Tutorial (CFPT) computer-based pro- gram providing multimedia training and feedback regarding food portions of common food items.<br>• Immediate training (IT) - Participants received CFPT training before estimating portion sizes of a buffet meal.<br>• Delayed training (DT)- Received CFPT training following the meal and were then asked to re-estimate portions following training.	Assess if web-based food portion training program (CFPT) improves portion size estimation accuracy.	Adult-Both sexes<br>18-older (52± 15 y)<br>OW, O<br>Healthy<br>n=76	CFPT program modulated and improved the variation/error between the estimated and weighed portions however it failed to improve accuracy in the estimation; training exposure shift underestimation to overestimation.<br>Participants rated the program on 5-point Likert scale as useful (4.1 ± 0.8) and easy to use (4.5 ± 0.9).
Rolls et al. 2017<br><br>*USA.*	Three-arm randomized controlled trial<br>12 months	***<u>SET</u>***<br>***<u>Non-Tw- Comp. Tools</u>***<br>• Pre-portioned main dishes daily for lunch and dinner (individual servings of main dishes, side dishes, snacks, yogurt, and whole fruits).<br>• Portion Selection Tools set (Digital food scale; measuring cups and spoons; placemat illustrating appropriate proportions of meal components; Portion size card with common objects).<br>• Standard advice - Educational sessions and materials (control)	Evaluate if the use of portion control tools or pre-portioned food would promote more weight loss than following standard advice.	Adult-Both sexes<br>20-65 y<br>OW, O-I, II<br>Healthy<br>N=186 (62 per arm)	The tool set and guidelines helped reduce energy density of the dietbut there were no significant differences in body weight at 6 or 12 months compared with the standard advice or the pre-portioned group (alternative intervention, which was the most effective at 3 months).<br>Adherence - general indicators not significantly different across groups.<br>Satisfaction ratings with diet differed across the groups. Pre-portioned Foods Group reported spending less time and effort on meals and feeling more self-conscious and deprived when eating (no change over time on diet satisfaction for the other two groups).

PS Choice					
Almiron-Roig et al. 2016; 2019<br><br>*UK*	Randomized crossover trial including a qualitative sub-study<br><br>4 weeks (2 weeks with each tool)	***SET***<br>• Set of calibrated crockery (plate, bowl, glass)<br>• Set of plastic serving spoons (CHO, PRO, FV)	Acceptability and pattern of use of two portion control sets (serving utensils and tableware) by individuals with obesity	Adult-Both sexes<br>Not specified (Age)<br>OW, O<br>Healthy<br>n= 29	Both tools were perceived to be effectiveness easy to use, well accepted (all comparisons p>0.05), and as practical strategies for portion size knowledge.<br>Using both tools increased vegetables (raw and cooked) self-selected portions size and decreased potatoes and chips (self-reported data).<br>No significant differences were found on tool acceptance across tool types. All tools were reported as ease to use, well accepted and effective.
Di Santis et al 2013<br><br>*USA*	Randomized crossover trial<br>8 days (school lunch)	***Tw-Eating Plate sizes***<br>• Child-size Plate (7.3”) and bowl (8 oz.)<br>• Adult-size Plate (10.3”) and bowl (16 oz.) diameter plate and side 8-oz bowl.	Evaluate the effect of dishware size on young children self-served portion sizes and energy intakes at school lunch.	Children-Both Sexes<br>Age: 5-6 y<br>Not Specified (Health status)<br>n= 42	Chid-size dishware reduced self-served PSs when compared to adult-size dishware (p<0.01). Food liking and meal format (unit entrée) enhanced this effect. Children self-served 239 kcal more when the unit entrée was offered (p<0.001). When they liked the entrée, children served themselves 104 kcal more energy at the meal (p<0.001).
Fisher et al. 2013<br><br>*USA*	2 x 2 Randomized crossover trial	*Tw-Serving Spoon Size*<br>• Serving spoon sizes: tablespoon and teaspoon<br>• Amount of entrée available: 275g and 550g	Evaluate the effect of the amount of food available and spoon serving size.	Children-Both Sexes<br>Age: 4-6 y<br>Not specified (Health status)<br>n= 60	Teaspoons reduced entrée serving size by 11.5% (Tablespoon increased serving sizes by 13% compared to teaspoon (p<0.05)).<br>Amount of food served in grams (Mean ±SD) :<br>• Teaspoon: 83.7 ± 14.7<br>• Tablespoon: 73.8 ± 14.7<br>When children were exposed to the large size entrée (550 g), they self-served 40% (p<0.001) more food than with the small (275 g).<br>Served amount in grams (Mean ± SD):<br>• Small entrée size (272 g): 65.6 ± 14.7<br>• Large entrée size (550 g): 91.9 ± 14.7
Hughes et al. 2017<br><br>*USA*	Two randomized crossover trials<br>1 day	***Tw-Eating Plate sizes***<br>• 3D plate (21 cm) with indicators for grain/starches, PRO and FV.<br>• Regular plate (30 cm)<br>With USDA guidelines (Study 1) or household measure guidelines (Study 2)	Determine the efficacy of the use of a novel portion control plate in on serving portion sizes and intake.	Adult-Both sexes<br>Not Specified (Age)<br>NW, OW, O-I<br>Healthy<br>n=70 (Study 1)<br>n=40 (Study 2)	Calibrated plate produced smaller self-selected portion sizes of all foods in both studies. However, vegetables serving sizes were smaller than recommended on both portion control and regular plate.
Koh and Pliner 2009 (Study 4)	Mixed-methods randomized controlled trial	***Tw-Eating Plate sizes***<br>• Large Plate (23.5 cm)<br>• Small Plate (18.2 cm)	Study the effects of three social/environmental variables ((friends vs.	Adult-Women<br>17-26 y (19.2 y)<br>NW, OW, O-I, II	The small plate reduced served amount in the sharing condition, but no significant difference was detected (p>0.05) This effect was not found with the larger plate. Moreover,

Canada	(cross-over and parallel)<br>1 day	• Serving bowl, non-shared<br>Serving bowl, shared	strangers, plate size (large vs. small), and sharing (sharing vs. non-sharing)) on eating behaviour.	Healthy<br>n=57	participants in the sharing (eating with a friend) self-served more food than eating with strangers (non-sharing condition), showing an effect of acquaintance (*p*<0.01).<br><br>Small plate served amount in grams (Mean ± SD):<br>• Friends no share: 551.7 ± 140.6<br>• Friends share: 387.3 ± 63.6<br>• Strangers no share: 411.7 ± 87.5<br>• Strangers share: 319.2 ± 64.1
Kroeze et al. 2018<br><br>*Netherlands*	Observational Study<br>9 months	***<u>Non-Tw- Comp. Tools</u>***<br>Web based *PortionSize@warenessTool* (educational on-line program consisting on a digital dish-up for poerion size knowledge and awareness) as part of a combined educational intervention consisting of two phases (3 and 9 months)[4] (**SMARTsize**)	Enhance an adequate portion control behaviour to decrease energy intake and body weight.	Adult-Both sexes<br>(41.8 ± 9.9) y<br>OW, O-I,II<br>Healthy<br>n=225	Self-reported strategies to control food portion size significantly improved after 3 months of intervention (*p* < 0.001) (e.g. prepare low- calorie dishes, intention to consume smaller portions and the use of portion control strategies). In addition, an improvement of self-reported BMI was reported from baseline to 9 months (*p* < 0.001).<br>Individual counselling had no impact on outcomes.<br>Almost all participants (90%) reported they used the website and were satisfied with the intervention (grade between 7.2 and 8.0 on a 10-point scale).
Libotte et al. 2014<br><br>*Switzerland*	Parallel randomized controlled trial (fake buffet)<br>1 day	***<u>Tw-Eating Plate+Bowl sizes</u>***<br>Dishware sizes:<br>• Standard Plate (27 cm), bowl (14 cm), plate (16 cm)<br>• Large plate (32 cm), bowl (14 cm), plate (16 cm)	Investigate plate size influence on food choices of normal eaters in a self-service setting (fake food buffet)	Adult-Both sexes<br>Mean: 24.5 ± 4.3 y<br>NW<br>Healthy<br>n=83	Plate size did not have an effect on total energy of the meal (*p*>0.05).<br>Large plate induced to serve significantly larger vegetables portion sizes (*p*<0.05).
Rollo et al. 2017<br><br>*Australia*	Three-arm randomized controlled trial	***<u>Non-Tw- Comp. Tools</u>***<br>• *ServAR technological tool* (Augmented reality educational tool to guide the serving of food for portion control (tablet).<br>• Control group (no intervention)	Evaluate ServAR's (Augmented reality tool to guide the serving of food for portion control (tablet)) usability, impact (accuracy) on estimation of standard food serving sizes and commonly consumed foods.	Adult-Both sexes<br>18-35 y (25.8 (24.9 – 26.7)<br>NW, OW<br>Healthy<br>n= 90	ServAR improved the accuracy and consistency of estimated portion size when compared to the other two groups. The app increased the number of foods for which the estimated amounts matched reference portion size and reduced error rate vs. reference.<br>ServAR tool was perceived easy to use and well accepted.

[4] n=66 (online program, cooking lessons and other strategies during 3 months); n=159 (9 months, the same as the 3-month group but with individual counseling).

Van Kleef et al. 2012<br><br>*USA*	Parallel randomized trial<br>1 day	***Tw- Serving Dish sizes***<br>• Large serving bowl (6.9 L)<br>• Medium serving bowl (3.8 L)	Determine the impact of serving bowls size on food serving behaviour and food intake.	Adults-Both sexes (Undergraduate students)<br>20.5 ± 2.4 y<br>NW<br>Healthy<br>n=68	Large-sized bowl diners served 77% more pasta and consumed 71% more when compared to the medium-sized bowl ($p< 0.05$). Bowl size effect was reported on amount of food served was significant ($p<0.01$).<br>Satiation levels were higher with the large-size bowl compared to the medium-size ($p<0.01$)<br>Pasta served in kcal (Mean ± SD):<br>• Medium-sized Bowl: 205.5 ± 69.6<br>• Large-sized Bowl: 364.0 ± 139.9
Van Ittersum and Wansink 2013<br><br>*USA*	Randomized cross-over trial<br>4 days (school)	***Tw-Eating Bowl sizes***<br>• Large Bowl (16 oz)<br>• Small Bowl (12 oz)	Examine the impact of extraversion on serving behaviour using two different bowl sizes.	Children-Both sexes classed as extraverted or introverted<br>6-12 y (8.3)<br>Not Specified (BMI)<br>Healthy<br>n= 18	Small bowl reduced self-served amount of cereal by 44%, especially for extraverted children.<br>When children served themselves, they served and consumed 23.2% more cereal than when an adult served themandeemed to be more sensitive to bowl size when they self-served. On the contrary, when adults became involved, introverted children became more sensitive to the size of bowl but the interaction between bowl size and serving person was not significant ($p>0.05$).
Wansink et al. 2006<br><br>*USA*	Parallel semi-randomized trial<br>1 day<br>(professional social event)	***Tw-Eating Bowl+Spoon sizes***<br>• Small Bowl (17 oz) with small (2 oz) or large (3 oz) ice-cream scoop.<br>• Large Bowl (34 oz) with small (2 oz) or large (3 oz) ice-cream scoop.	Examine the impact of bowl and serving spoon size on experts (graduate students, and staff members of the Department of Food Science and Nutritional Science of a large Midwestern University) serving sizes and food intake.	Adult-Both sexes (Nutrition experts)<br>Not Specified (Age)<br>Not Specified (BMI)<br><br>n=85	The small-sized bowl reduced 24% the self-served ice cream portion size. Experts who received a larger bowl served and ate 31 % more ice cream than those who received a smaller bowl (Mean; 6.25 vs 4.77 oz.) ($p<0.01$), but they did not perceive themselves as having served more ($p>0.05$).<br><br>When the serving spoon size was increased 50%, regardless of bowl size, participants served and ate 12.7% more ice cream. The influence of spoon size was most notable when combined with a large bowl.
PS Intake					
Ahn et al. 2010<br><br>*Korea*	Randomized crossover trial<br>3 months (at home)	***Tw- Eating Bowl sizes***<br>• Small bowl (200 mL), Regular bowl (380 mL)	Evaluate the effect rice bowls sizes on total energy intake, carbohydrate intake, body weight, and blood glucose levels.	Adult-Women<br>20-70 y (55.1 ± 7.1)<br>NW, OW, O<br>DMT2<br>n=42	The small bowl reduced total energy intake ($p<0.001$), carbohydrate intake, body weight, and blood glucose levels
Ayaz et al. 2016<br><br>*Turkey*	Randomized crossover trial<br>3 days (buffet)	***Tw-Eating Plate sizes***<br>• Large plate (28 cm)<br>• Medium plate (23 cm)<br>• Small plate (19 cm)	Examine the effect of plate size on energy and macronutrient intake.	Adult-Women<br>19-25 y (22.93 ± 2.83)<br>NW	No effect of the smaller plate size was report on reducing energy or specific macronutrient intake ($p>0.05$).

				Healthy<br>n=37	
Di Santis et al 2013<br><br>*USA*	Randomized cross-over trial<br>8 days (school lunch)	***Tw-Eating Plate sizes***<br>• Child-size Plate (7.3″) and bowl (8 oz.)<br>• Adult-size Plate (10.3″) and bowl (16 oz.) diameter plate and side 8-oz bowl.	Evaluate the effect of dishware size on young children self-served portion sizes and energy intakes at school lunch.	Children-Both Sexes<br>Age: 5-6 y<br>Not Specified (Health Status)<br>n= 42	Child-size dishware reduced total meal energy intake when compared to adult-size dishware (50.8 kcal difference; $p<0.001$). Adult-size dishware promoted energy intake indirectly ($p<0.001$). It induced an increase of 0.43 kcal consumed for each additional kcal served.
Fisher et al. 2013<br><br>*USA*	2 x 2 Randomized crossover trial	*Tw-Serving Spoon Size*<br>• Serving spoon sizes: tablespoon and teaspoon<br>Amount of entrée available: 275g and 550g	Evaluate the effect of the amount of food available and spoon serving size.	Children-Both Sexes<br>Age: 4-6 y<br>Not specified (Health Status)<br>n= 60	No effect of spoon size on food intake.<br>Children who selected more of the entree tended to eat more of this ($p<0.0001$) and to consume greater energy at the meal ($p<0.0001$).
Koh and Pliner 2009 (Study 4)<br><br>*Canada*	Mixed-methods randomized controlled trial (cross-over and parallel)<br>1 day	***Tw-Eating Plate sizes***<br>• Large Plate (23.5 cm)<br>• Small Plate (18.2 cm)<br>• Serving bowl, non-shared<br>• Serving bowl, shared	Study the effects of three social/environmental variables ((friends vs. strangers, plate size (large vs. small), and sharing (sharing vs. non-sharing)) on eating behaviour.	Adult-Women<br>17-26 y (19.2 y)<br>NW, OW, O-I, II<br>Healthy<br>n=57	The small plate reduced intake amount in the sharing condition ($p<0.05$) This effect was not found with the larger plate. Moreover, participants in the eating with friends self-serve more food than eating with strangers (non-sharing condition), showing an effect of acquaintance ($p<0.01$).<br><br>Small plate consumed amount in grams (Mean ± SD):<br>• Friends no share: 534.2 ± 145.1<br>• Friends share: 387.3 ± 63.6<br>• Strangers no share: 407.2 ± 89.9<br>• Strangers share: 312.9 ± 68.9<br><br>Participants ate almost all the food they have self-served (significant correlation; $p<0.001$)
Kosite et al. 2019<br><br>*UK*	Parallel randomized controlled trial<br>1 day	***Tw-Eating Plate sizes***<br>• Large Plate (29 cm)<br>• Small Plate (23 cm)	Examine the impact of plate size on served and consumed food and meal micro-structure (number of servings and eating rate).	Adult-Both sexes<br>18-60 y<br>OW, O<br>Healthy<br>n= 134 (67 per group)	No effect of plate size was reported on total energy or eating parameter ($p>0.05$). Mean difference on the total intake between both plates (small vs. large) was 19.2 kcal, showing no significant difference and a very small size effect ($d = 0.07$). Group differences were found only in one meal microstructure measure; leftovers. Participants using larger plates left 8.6 g more of food after the meal than participants who used the smaller plate.

Mishra et al. 2012<br><br>*USA*	Parallel trials (field study and controlled lab setting)<br><br>1 d	*Tw-Eating Cutlery*<br>• Small fork (20% less capacity than regular fork)<br>• Large fork (20% more capacity)	Evaluate the effect of the amount of food available and fork size.	Adults<br>Not Specified (Age)<br>Not Specified (BMI)<br>Not Specified (Health Status)<br>Lab study (n=81)	Smaller fork increased food consumption compared to the larger fork when used in restaurant setting, i.e. more food was left on the plate with the large (average 7.9 oz) vs. small fork (average 4.4 oz) ($p<0.01$).<br><br>Opposite pattern was found in the lab where pasta consumption was decreased with the small fork, i.e. less food was left on the plate with the large (average 4.1 oz) vs. small fork (average 5.2 oz) ($p<0.05$).
Kroeze et al. 2018<br><br>*Netherlands*	Observational Study<br>9 months	***Non-Tw- Comp. Tools***<br>Web based *PortionSize@warenessTool* (educational on-line program consisting on a digital dish-up for poerion size knowledge and awareness) as part of a combined educational intervention consisting of two phases (3 and 9 months) [5]<br>(**SMARTsize**)	Enhance an adequate portion control behaviour to decrease energy intake and body weight.	Adult-Both sexes (41.8 ± 9.9) y<br>OW, O-I,II<br>Healthy<br>n=225	Self-reported strategies to control food portion size significantly improved after 3 months of intervention ($p < 0.001$) (e.g. prepare low- calorie dishes, intention to consume smaller portions and the use of portion control strategies). In addition, an improvement of self-reported BMI was reported from baseline to 9 months ($p < 0.001$).<br>Individual counselling had no impact on outcomes.<br>Nearly 90% of the participants reported they used the website and were satisfied with the intervention (grade between 7.2 and 8.o on a 10-point scale).
Pilling et al. 2020<br><br>*UK*	Mega-analysis	Wine glasses size bars:<br><br>• Size 4 (450 ml)<br>• Size 3 (370 ml)<br>• Size 2 (310 ml)<br>• Size 1 (250 ml)<br>Wine glasses size restaurants:<br><br>• Size 4 (510 ml)<br>• Size 3 (450 ml)<br>• Size 2 (370 ml)<br>• Size 1 (250 ml)	Provide robust information of the effect of wine glass size on volume of wine sold in bars and restaurants.	Adult- Sex Not Specified<br>Not Specified (Age)<br>Not Specified (BMI)<br>Not Specified (Health Status)	Mega-analysis showed that when comparing bar sales of different glass sizes to the standard size (300 ml), only 450 ml glasses showed higher sales.<br>Wine sales:<br>370-ml glasses was 0.5% lower (95% CI = –8.1% to 6.1%)<br>450-ml glasses was 1.0% higher (95% CI = –9.1% to 12.2%)<br>510-ml glasses was 0.4% lower (95% CI = –9.4 to 9.4).<br>For restaurants, the effect was detected on 370 ml glass, increasing 7.3% wine sales when compared to 300 ml glass.<br><br>Wine sales:<br>250-ml glasses was 9.6% lower (95% CI = –19.0 to 0.7).<br>370-ml glasses was 7.3% higher (95% CI = 1.5% to 13.5%)<br>450-ml glasses was 0.9% higher (95% CI = –5.5 to 7.7).

[5] n=66 (online program, cooking lessons and other strategies during 3 months); n=159 (9 months, the same as the 3-month group but with individual counseling).

.

Robinson et al. 2015<br><br>*UK*	Parallel randomized controlled trial<br><br>1 day	***Tw-Eating Bowl sizes***<br>• Large bowl (18 cm)<br>• Small bowl (16 cm)	Evaluate the effect of bowl size on snack food consumption	Adult-Both sexes<br>19-59 y (31.2 ± 9.8)<br>NW, OW<br>Healthy<br>n=61<br>• Large bowl (n=30)<br>• Small bowl (n=31)	No bowl size effect size was detected on food intake, although the small bowl favoured higher food consumption when compared to the large bowl (p>0.05).
Rolls et al. 2007 (Study 1)<br><br>*USA*	Randomized crossover trial<br>3 days	***Tw-Eating Plate sizes***<br>• Large Plate (26 cm)<br>• Medium Plate (22 cm)<br>• Small Plate (17 cm)<br>1 course, self-selected PS	Examine the effect plate size on energy intake.	Adult-Both sexes<br>20-45 y<br>NW, OW, O-I, II<br>Healthy<br>n=45	Plate size had no significant effect on meal energy intake (p>0.05).<br>The mean ± SD differences intake using the smallest and largest plates was of 21±13 g, equivalent to < 142 kJ (34 kcal) and not significantly different from zero.
Rolls et al. 2007 (Study 2)<br><br>*USA.*	Randomized crossover trial<br>2 days	***Tw-Eating Plate+Spoon sizes***<br>• Large Plate (26 cm cm) and soup spoon (50% larger than the standard)<br>• Medium Plate (22 cm) and standard spoon<br>1 course, fixed PS	Test the effect of plate size and eating utensils on both the perception of the amount of food on the plate and food intake	Adult-Both sexes<br>20-45 y<br>NW, OW, O-I, II<br>Healthy<br>n= 30	The size of the plate or spoon (p>0.05) had no impact on energy intake irrespective of subject characteristics. Mean differences in intake using the smallest and largest plates was of 11±13 g (mean ± SD) equivalent to < 142 kJ (34 kcal).<br>Most (80%) participants correctly stated that the amount of food on both plates were the same while 20% perceived them as different.
Rolls et al. 2007 (Study 3)<br><br>*USA.*	Randomized crossover trial<br>3 days	***Tw-Eating Plate sizes***<br>• Large Plate (26 cm)<br>• Medium Plate (22 cm)<br>• Small Plate (17 cm)<br>Buffet, self-selected PS	Evaluate the effect of plate size on food intake.	Adult-Both sexes<br>20-45 y<br>NW, OW, O-I, II<br>Healthy<br>n= 44	No plate size effect was reported on meal energy intake (*p* >0.05). Smaller plate led to a larger number of trips to the buffet compared to the other two plate-sizes.<br>Intake mean difference ± SD when comparing the smaller vs. the largest plate was of 4±18 g, (34 kcal).
Rolls et al. 2017<br><br>*USA.*	Three-arm randomized controlled trial<br>12 months	***SET***<br>***Non-Tw- Comp. Tools***<br>• Pre-portioned main dishes daily for lunch and dinner (individual servings of main dishes, side dishes, snacks, yogurt, and whole fruits).<br>• Portion Selection Tools set (Digital food scale; measuring cups and spoons; placemat illustrating appropriate proportions of meal components; Portion size card with common	Evaluate if the use of portion control tools or pre-portioned food would promote more weight loss than following standard advice.	Adult-Both sexes<br>20-65 y<br>OW, O-I, II<br>Healthy<br>N=186 (62 per arm)	There was no impact of any intervention on body weight at 6 or 12 months compared to control. Pre-portioned foods group had a greater rate of initial weight loss but this effect was lost over time.<br>The tool set and guidelines helped reduce dietary energy density. The Portion selection group was the only group that maintained food intake, on the contrary, food intake decreased by 11% on the other two groups (interaction *p*<0.01).<br>Adherence general indicators were not significantly different across groups.<br>Satisfaction ratings with diet differed across the groups. Pre-portioned Foods Group reported spending less time and effort on meals and feeling more self-conscious and deprived when eating. The other two groups did not report any change over

		objects).<br>• Standard advice - Educational sessions and materials (control).			time on diet satisfaction.
Shah et al. 2011<br><br>*USA*	Parallel randomized controlled trial<br>2 days	***Tw-Eating Plate sizes***<br>• Small plate (22 cm)<br>• Large plate (27 cm)	Examine the effect of plate size on energy intake in women with overweight/obesity and normal weight.	Adult-Women<br>45 ± 14.6 y<br>NW, OW, O-I,II<br>Healthy<br>n= 20	No plate size effect was detected, regardless of weight status, on the amount of energy consumed, taste of the menu, satiety or subjective appetite (*p*>0.05).<br><br>Energy intake normal weight in kJ (Mean ± SD):<br>• Small plate: 1356 ± 515<br>• Large plate: 1365 ± 393<br><br>Energy intake overweight in kJ (Mean ± SD):<br>• Small plate: 1314 ± 632<br>• Large plate: 1226 ± 431
Shimpo & Akamatsu. 2018<br><br>*Japan*	Randomized crossover trial<br>1 day	Bowl Size<br>• Large Bowl (13.5 cm)<br>• Small Bowl (11.5 cm)<br>Rice Portion Size<br>• Small portion size (150 g)<br>• Large rice portion size (250 g)	Evaluate the effect of portion size and bowl size.	Adult<br>20-60 y ()<br>NW<br>Healthy<br>n=21	Portion size showed a significant effect on rice consumption. Larger portion size promoted higher rice intake than the exposure to small rice portion size (p<0.001). No interaction of bowl size was detected (p>0.05).
Vakili et al 2019<br><br>*Iran*	Parallel randomized controlled trial<br>2 days	***Tw-Eating Plate+Spoon sizes***<br>Ceramic/glass tableware:<br>• Large plate, spoon and fork (25 cm; 15 ml); glass 120 ml<br>• Small plate, spoon and fork (19.5 cm; 5 mL); glass 120 ml	Study the effect of plate, spoon and fork size on meal intake of participants with overweight.	Adult-Both sexes (Clerical staff of the university)<br>19-50 y (39 ± 6.98)<br>OW, O-I,II<br>Healthy<br>n=40	The small tableware reduced rice consumption, but no effect was found on total energy intake.<br><br>The only food for which significant differences were detected when comparing small v. large utensils was the rice intake with large utensil set (*p*<0.05).<br>Consumed Rice in g (Mean ± SD):<br>• Small utensils: 206.8 ± 71.1<br>• Large utensils: 235.4 ± 86.3<br><br>Grain products consumption but no other meal component changed with changing in dishware size with no effect on total energy intake.
Van Kleef et al. 2012<br><br>*USA*	Parallel randomized trial<br>1 day	***Tw- Serving Dish sizes***<br>• Large serving bowl (6.9 L)<br>• Medium serving bowl (3.8 L)	Determine the impact of serving bowls size on food serving behaviour and food intake.	Adults-Both sexes (Undergraduate students)<br>20.5 ± 2.4 y<br>NW	Large-sized bowl diners served 77% more pasta and consumed 71% more when compared to the medium-sized bowl (*p*< 0.05). Satiation levels were higher with the large-size bowl compared to the medium-size (*p*<0.01)

				Healthy<br>n=68	Pasta consumed in Kcal (Mean ± SD):<br>• Medium-sized Bowl: 176.7 ± 72.8<br>• Large-sized Bowl: 301.3 ± 129.6
Venema et al. 2020<br><br>*Netherlands*	Mixed-methods randomized trial (Crossover for spoon size and parallel for habit context condition)<br>2 days	***Tw- Serving Spoon sizes***<br>• Large spoon (5ml)<br>• Small spoon (2.5 ml)<br><br>Both spoon size with either habit context disruption (cold tea) or habit context preservation (hot tea).	Evaluate the effect of spoon size and habit disruption context on sugar added to tea.	Adults-Both sexes<br>21.42 ± 4.09 y<br>Not Specified (BMI)<br>Healthy<br>n=123	Participants consumed less sugar added to the tea (27%) with the small-size spoon (*p*<0.001).<br>This effect was reduced in people who had a stronger habit of adding a fixed amount of sugar to the tea.<br>No significant effect was found either on temperature condition or habit strength.<br><br>Added sugar in grams (Mean ± SD):<br>• Normal-sized spoon: 5.13 ± 4.81<br>• Small-sized spoon: 3.50 ± 2.64
Wansink and Van Ittersum 2013 (Study 2)<br><br>*USA*	Observational Study<br>1 day (Chinese restaurant-buffet)	***Tw-Eating Plate sizes***<br>• Smaller plate (25 cm)<br>• Larger plate (29 cm)	Examine if portion norms influence serving sizes and consumption in a natural eating environment (Chinese restaurant).	Adult-Both sexes<br>18-80 y (40.7)<br>OW<br>Not specified (Health Status)<br>n=43	When eating with the larger plate, diners served 45% more than total food those who used the small plate (1,216.9 vs. 800.5 cm^2; *p*<0.01).<br>Participants that received smaller plates reduced total energy intake by 31% and leftovers by 42%. The effect could be partly influenced by baseline hungers levels.
Wansink et al. 2006<br><br>*USA*	Parallel semi-randomized trial<br>1 day (professional celebration)	***Tw-Eating Bowl+Spoon sizes***<br>• Small Bowl (17 oz) with small (2 oz) or large (3 oz) ice-cream scoop.<br>• Large Bowl (34 oz) with small (2 oz) or large (3 oz) ice-cream scoop.	Examine whether the size of a bowl or serving spoon unknowingly biases how much a person/expert (graduate students, and staff members of the Department of Food Science and Nutritional Science of a large Midwestern University) attending a social event	Adult-Both sexes (Nutrition experts)<br>Not Specified (Age)<br>Not Specified (BMI)<br>Not Specified (Health Status)<br><br>n=85	The experts who received a larger bowl served and ate 31.0% more ice cream than those who received a smaller bowl (6.25 vs 4.77 oz.) (*p*<0.01).<br>When the serving spoon size was increased 50%, regardless of bowl size; participants served and ate 12.7% more ice cream.<br>The influence of spoon size was most notable when combined with a large bowl.<br>Although smaller spoons increased the number of tablespoons, consumption amount did not increased.
Wansink et al. 2014 (Study 1)<br><br>*USA*	Parallel randomized controlled trial<br>1 day (schools)	***Tw-Eating Bowl sizes***<br>• Small bowl (8 oz)<br>• Large bowl (16 oz)	Investigate the effect of bowl size on children food request and serving sizes.	Children-Both sexes<br>4.1± 0.65 y<br>OW<br>Healthy<br>n=69	Small bowl reduced the amount of cereal requested by children (served by adults) by 47% compared to the large bowl (*p*<0.001).<br>Cereal serving sizes (small bowl vs. large bowl) (Mean ± SD) : 24.7±14.9 g vs. 46.1±15.1 g.

Wansink et al. 2014 (Study 2)<br><br>*USA*	Randomized crossover trial<br>2 days<br>(summer camp)	***Tw-Eating Bowl sizes***<br>• Small bowl (8 oz)<br>• Large bowl (16 oz)	Investigate the effect of bowl size on children serving size and food intake. .	Children-Both sexes (low-income families)<br>6-12 y<br>Healthy<br>Low SES<br>n=18	The small bowl reduced cereal and milk amount self-selected (served by adults) by 41% compared to the large bowl (average 207.1 g vs. 349.9 g; p<0.01). The effect was maintained after subtracting leftovers.
Yip et al. 2013<br><br>*New Zealand*	Randomized crossover trial<br>2 days	***Tw-Eating Plate sizes***<br>• Small plate (20 cm)<br>• Large plate (27 cm)	Test the effect of plate size on energy intake.	Adult-Women<br>20-51 y<br>OW, O-I,II<br>Healthy<br>n=20	No plate size effect was reported in either total energy or macronutrient consumption (buffet with attractive foods) (p>0.05).<br>Mean intake ± SEM when participants used the smaller plate was: 3975 ± 239 kJ (range 2113-5674 kJ)<br>Mean intake ± SEM when participants used the larger plate was: 3901 ± 249 (range 1748-6284 kJ)
Weight Status					
Ahn et al. 2010<br><br>*Korea*	Randomized crossover trial<br>3 months (at home)	***Tw- Eating Bowl sizes***<br>Small bowl (200 mL), Regular bowl (380 mL)	Evaluate the effect rice bowls sizes on total energy intake, carbohydrate intake, body weight, and blood glucose levels.	Adult-Women<br>20-70 y (55.1 ± 7.1)<br>NW, OW, O<br>DMT2<br>n=42	Both groups reported significant reductions on body weight after 12 weeks. No significant differences were found among groups.
Ho et al. 2016<br><br>*Canada*	Parallel randomized controlled trial<br>6 months<br>(families)	***Tw-Eating Calib. Plate+Bowl***<br>• Calibrated Tableware (*Diet Plate*- plate and bowl) + nutritional counselling (**FOCUS** intervention)<br>Control: Standard care nutritional counselling	Effect of a family intervention using a portion control tool on BMI z score in children with overweight and obesity.	Children-Both sexes<br>Age: 8-16 y (11 ± 2.2)<br>O<br>Healthy<br>n= 99<br>• Intervention (n=51)<br>Control (n=48)	No effect of the tableware was found on BMI z-score. Reduction in BMI z-score between baseline and 6 months was reported in both groups, but no effect of tableware was found.
Huber et al. 2015<br><br>*USA*	Parallel randomized controlled trial<br>6 months<br>(Mayo Clinic)	***Tw-Eating Calib. Plate***<br>• Calibrated tableware (transparent glass with guidelines and text- plate and bowl) and tele-coaching.<br>Usual care	Evaluate the impact of tele-coaching combined with a portion control plate on weight loss, BMI and waist circumference of patients with obesity.	Adult-Both sexes<br>18-70 years old<br>O-I, II<br>Healthy<br>• Intervention (n=45)<br>Control (n=45)	The combined use of tele-coaching and calibrated tableware showed significant reductions on body weight, BMI, and hip-waist ratio. These differences depend upon sex. Intervention reduced women body weight and BMI at 3 months (both p<0.05). The effect did not persist at 6 months.<br>A reduction in the hip-waist ratio was only detected in men at 3 months (p<0.05).

Jayawardena et al. 2019<br><br>Sri Lanka	Parallel Randomized controlled trial<br>3 months	***Tw-Eating Calib. Plate***<br>• Calibrated plate (printed indicators) (10.5") divided into 5 segments (rice, PRO and 3 types of vegetables)<br>Standard cardiac rehabilitation (no plate).	Evaluate the effectiveness of a plate model as part on dietary modification of cardio metabolic risk factors in overweight patients with MI (myocardial infraction) history.	Adult-Both sexes<br>20-70 y (50.5± 10.6)<br>NW,OW, O-I<br>Acute coronary syndrome<br>n=79<br>• Intervention (n=40)<br>Control (n=39)	Calibrated plate reduced BMI at 3 months of intervention compared with the control condition (*p*<0.05).<br><br>Weight loss in kg (Mean ± SD)<br>• Intervention: -1.27 ± 3.58 kg?<br>• Standard care: -0.26 ± 2.42 kg?<br>Weight loss was significantly higher in patients with overweight and obesity than those with normal weight (*p*<0.05).
Kesman et al. 2011<br><br>*USA*	Parallel randomized controlled trial<br>6 months<br>(Mayo Clinic)	***Tw-Eating Calib. Plate+Bowl***<br>• Calibrated tableware (transparent glass with guidelines and text- plate and bowl) and dietary counselling.<br>Usual care	Evaluate the effectiveness of portion control intervention (dietary counselling and portion control plate) to promote weight loss on patients with obesity, when compared to usual care.	Adult-Both sexes<br>18-75 y (55.9 ± 10.0)<br>O-II, O-III<br>Healthy<br>• Intervention (n=33)<br>• Control (n=32)	Intervention including the calibrated plate induced greater weight loss compared to the conventional treatment at 3 months (*p*<0.05). This effect did not persist at 6 months.<br><br>Weight loss percentage (Mean ± SD)<br>• Intervention: -2.4 ± 3.7%<br>• Usual care: -0.5 ± 2.2 %<br><br>Almost 50% of participants perceived that the intervention was helpful and 42% would continue using the portion control plate after the study . Thirty-two percent reported that they used the plate for one meal per day; 37% for two meals and 26% for three meals per day.
Kroeze et al. 2018<br><br>*Netherlands*	Observational Study<br>9 months	***Non-Tw- Comp. Tools***<br>Web based<br>*PortionSize@warenessTool*<br>(educational on-line program consisting on a digital dish-up for poerion size knowledge and awareness) as part of a combined educational intervention consisting of two phases (3 and 9 months[6])<br>(**SMARTsize**)	Enhance an adequate portion control behaviour to decrease energy intake and body weight.	Adult-Both sexes<br>(41.8 ± 9.9) y<br>OW, O-I,II<br>Healthy<br>n=225	An improvement of self-reported BMI was reported from baseline to 9 months (*p* < 0.001).<br>Individual counselling had no impact on outcomes.<br>Nearly 90% of the participants reported they used the website and were satisfied with the intervention (grade between 7.2 and 8.o on a 10-point scale).

[6] n=66 (online program, cooking lessons and other strategies during 3 months); n=159 (9 months, the same as the 3-month group but with individual counseling).

.

Pedersen et al. 2007<br><br>*Canada*	Parallel randomized controlled trial<br>6 months (Private clinic)	***<u>Tw-Eating Calib. Plate+Bowl</u>***<br>• Calibrated tableware (plate and bowl) with demarcations and illustrations (*The Diet Plate*); dietary assessment and book.<br>Usual care	Determine the efficacy of a portion control plate to induce weight-loss on patients with obesity and type 2DM.	Adult- Both sexes<br>37-77 y<br>O-I,II<br>DM2<br>n= 130<br>• Intervention (n=65)<br>• Control (n=65)	The calibrated tableware significantly improved cholesterol (*p*<0.05) and blood pressure levels (*p*<0.001), reduced the use of hypoglycaemic medication (*p*<0.05) and facilitated weight loss (*p*<0.01) (at least 5% weight loss) when compared to the usual care.
Poelman et al. 2015<br><br>*Netherlands*	Parallel randomized controlled trial<br>12 months	***<u>Non-Tw- Comp. Tools</u>***<br>Web based *PortionSize@warenessTool* (educational on-line program consisting on a digital dish-up for portion size knowledge and awareness) as part of a combined educational intervention (**PortionControl@HOME**)<br>Control group: wait-list control group	Evaluate the effect of using an interactive web-based multi-component educational tool on body mass index and control behavior.	Adult-Both sexes<br>18-60 y (45.65 ± 9.20)<br>OW, O<br>Healthy<br>n=278<br>• Intervention (n=139)<br>• Control (n=139)	Mean BMI decreased in both groups between baseline and 3 months. These differences were not maintained at 6 and 12 months but mean body mass index remained below the baseline values.<br>Almost all participants (90%) logged on to the website. Different adherence levels were reported for each intervention items (cooking classes, read educational book, filled-out the Home-Screener completely, etc.)
Rolls et al. 2017<br><br>*USA.*	Three-arm randomized controlled trial<br>12 months	***<u>SET</u>***<br>***<u>Non-Tw- Comp. Tools</u>***<br>• Pre-portioned main dishes daily for lunch and dinner (individual servings of main dishes, side dishes, snacks, yogurt, and whole fruits).<br>• Portion Selection Tools set (Digital food scale; measuring cups and spoons; placemat illustrating appropriate proportions of meal components; Portion size card with common objects).<br>Standard advice - Educational sessions and materials (control).	Evaluate if the use of portion control tools or pre-portioned food would promote more weight loss than following standard advice.	Adult-Both sexes<br>20-65 y<br>OW, O-I, II<br>Healthy<br>N=186 (62 per arm)	There was no impact of any intervention on body weight at 6 or 12 months compared to control. Pre-portioned foods group had a greater rate of initial weight loss but this effect was lost over time.<br>General indicators for adherence were not significantly different across groups.<br>Pre-portioned Foods Group reported spending less time and effort on meals and feeling more self-conscious and deprived when eating. The other two groups did not report any change over time on diet satisfaction.

Table S2. ROB -Risk of bias analysis (adapted from Cochrane guidelines)[7].

Abbreviations: ITT, intention-to-treat (every randomized subject was included in the analyses - completers and non-completers). WS, within-subjects or cross-over design (repeated measures).

Authors	Primary research question	Comparability between intervention & comparator	Participants awareness of study purpose	WS designs, consideration of order effects	WS designs, consideration for carryover effects	ITT analysis	Drop-out	Incomplete outcome reporting	Comments
Ahn et al. 2010	Low	High	High	N/A	N/A	Low	Low	Low	
Almiron-Roig et al. 2016; 2019	Low	High	High	N/A	N/A	High	High	Low	Participants had different degrees of weight loss experience
Ayaz et al. 2016	Low	Low	Low	Low	Low	Low	Low	Low	
Brown et al. 2019	Unclear	High	High	N/A	N/A	High	Unclear	Low	
Di Santis et al 2013	Low	Low	Low	Low	Low	Low	Low	Low	
Fisher et al. 2013	Low	Low	Low	Low	Low	High	High	Low	
Ho et al. 2016	Low	High	High	N/A	N/A	Low	High	Low	
Huber et al. 2015	Low	High	High	N/A	N/A	High	Low	Low	
Hughes et al. 2017	Low	Low	High	Low	Low	Unclear	Unclear	Low	*n* not reported in results
Jayawardena et al. 2019	Low	Low	High	N/A	N/A	High	High	Low	
Kesman et al. 2011	Low	High	High	N/A	N/A	Low	High	High	

[7] Higgins J, Thomas J, Chandler J, Cumpston M, Li T, Page M, et al. Cochrane Handbook for Systematic Reviews of Interventions Version 6.0 [updated July 2019]. London: The Cochrane Collaboration; 2019.

Koh and Pliner 2009 **(Study 4)**	Low	High	Low	N/A	N/A	Low	Low	Low	Friends vs. Strangers and sharing vs.not sharing conditions
Kosite et al. 2019	Low	High	Low	N/A	N/A	Unclear	Low	Low	Participants were filmed while eating
Kroeze et al. 2018	Low	High	High overt int	N/A	N/A	High	High	Low	
Libotte et al. 2014	Low	High	High	N/A	N/A	Low	Low	Low	Imitation food used
Mishra et al. 2012 (Restaurant study)	Low	High	Low	N/A	N/A	Unclear	Unclear	High	*n* not reported in results
Mishra et al. 2012 (Laboratory study)	Low	Low	High	N/A	N/A	Unclear	Unclear	High	Study described as "food consumption study"; Covariates not analysed
Pedersen et al. 2007	Low	High	High	N/A	N/A	Low	Low	Low	
Poelman et al. 2013	Unclear	Low	High	N/A	N/A	High	Unclear	Low	
Poelman et al. 2015	Low	High	High	N/A	N/A	Low	High	Low	
Pilling et al. 2020	Low	High	Low	N/A	N/A	N/A (sales data)	N/A (sales data)	Low	Wide range of restaurants and bars
Riley et al. 2007	Low	Low	High	N/A	N/A	Unclear	Unclear	Low	
Robinson et al. 2015	Low	Low	Low	N/A	N/A	High	Low	Low	Data for 2 subjects not analysed
Rollo et al. 2017	Low	Low**	Low	N/A	N/A	High	Low	Low	Overall unclear
Rolls et al. 2007 **(Study 1)**	Low	Low	Low	Low	Low	High	Low	Low	
Rolls et al. 2007 **(Study 2)**	Low	Low	Low Unclear	Low	Low	Low	Low	Low	

Rolls et al. 2007 **(Study 3)**	Low	Low	High	Low	Low	Low	Low	Low	
Rolls et al. 2017	Low	Low	Unclear	N/A	N/A	Low	Low	Low	
Shah et al. 2011	Low	Low	Low	Low	Low	Low	Low	Low	
Shimpo & Akamatsu 2018	Low	High	Low	Low	Low	Unclear	Unclear	Low	50% of subjects found rice PS much larger than usual however did not leave on plate for cultural reasons
Vakili et al. 2019	Low	Low	Unclear	Low	Low	Unclear	Low	Low	
Van Ittersum and Wansink 2013	High	High	Low	N/A	N/A	Unclear	Unclear	Low	Primary research question not clear
Van Kleef et al. 2017	Low	High	Low	N/A	N/A	Low	Low	Low	Participants ate with 3 more people
Venema et al. 2020	Unclear	Unclear	Unclear	Unclear	Unclear	High	Unclear	Unclear	Study design/methodolog y not clear
Wansink et al. 2006	Low	High	Low	N/A	N/A	N/A	N/A	Low	
Wansink and Van Ittersum 2013 **(Study 2)**	Low	High	Low	N/A	N/A	N/A	N/A	Low	Research made "ad hoc" with people happening to be
Wansink and Van Ittersum 2013 **(Study 3)**	Low	High	Low	N/A	N/A	N/A	N/A	Low	
Wansink et al. 2014 **(Study 1)**	Low	High	Low	N/A	N/A	N/A	Low	Low	
Wansink et al. 2014 **(Study 2)**	Low	High	Low	N/A	N/A	N/A	Low	Low	
Yip et al. 2013	Low	Low	High	Low	Low	Unclear	Low	Low	

Table S3. Egger´s test for funnel plot asymmetry. *Abbreviations*: FEMA, fixed-effects meta-analysis; REMA, random-effects meta-analysis.

Main Outcome	Number of comparisons	Hetero-geneity (RE) I^2	MA Random Effects Effect size (95%CI)	Egger's test (REMA) p-value	MA Fixed Effects Effect size (95%CI)	Egger's test (FEMA) p-value
Portion size choice	8	89.27%	-0.48 (-0.72, -0.24)	0.9574	-0.45 (-0.53, -0.38)	0.1734
Portion size intake[8]	21	89.02%	-0.22 (-0.38, -0.06)	0.3853	-0.19 (-0.24, -0.14)	0.0783
Portion size intake excluding Wansink et al. 2006 paper[9]	18	90.04%	-0.18 (-0.36, -0.01)	0.3861	-0.17 (-0.22, -0.11)	0.3753
Weight Status	9	84.40%	-0.20 (-0.37, 0.03)	0.0095	-0.13 (-0.20, -0.07)	0.0000

[8] Meta-analyses carried out with the small portion size condition in Shimpo & Akamatsu (2018). Results did not change when using the large PS instead (Egger´s test REMA 0.3676; FEMA 0.0593).

[9] Meta-analyses carried out with the small portion size condition in Shimpo & Akamatsu (2018). Results did not change when using the large PS instead (Egger´s test REMA 0.3639; FEMA 0.3020).

4. Supplementary Figures

Figure S1. Sensitivity analysis for studies examining the impact of portion control tools on portion size choice excluding data from Fisher et al. (2013)[10].

Forest plot for the analysis of all comparisons examining the effects of portion control tools on portion size choice (served amount) using random-effects meta-analysis. Contributing comparisons are represented by a filled square with horizontal lines, where the area of the square depicts the contribution of the study to the full analysis, and the horizontal lines indicate the 95% CIs for each study. Studies displaced to the left of a 0 line demonstrate a finding in favour of the portion control tool to reduce serving sizes, whereas those to the right demonstrate a finding in favour of the portion control tool promoting an increase on served amounts when compared with the control condition. The diamond at the base of the plot represents the combined effect (Standardized Mean Difference) with 95% CIs.

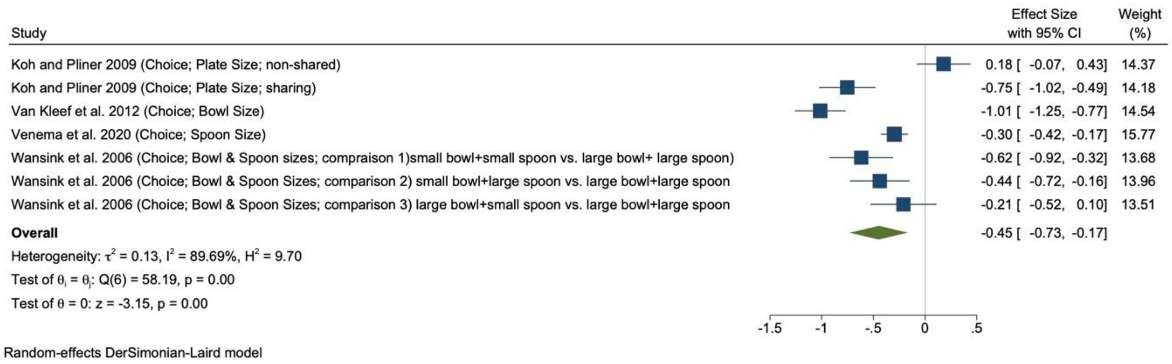

---

[10] Fisher JO, Birch LL, Zhang J, Grusak MA, Hughes SO. External influences on children's self-served portions at meals. Int J Obes. 2013;37(7):954–60.

Figure S2. Impact of portion control tools on portion size intake by study purpose awareness.

Forest plot for the analysis of all comparisons examining the effects of portion control tools on portion size intake (consumed amount) by study purpose awareness using a random-effects meta-analysis. Contributing comparisons are represented by a filled square with horizontal lines, where the area of the square depicts the contribution of the study to the full analysis, and the horizontal lines indicate the 95% CIs for each study. Studies displaced to the left of a 0 line demonstrate a finding in favour of the portion control tool to reduce food intake, whereas those to the right demonstrate a finding in favour of the portion control tool to increase consumed amounts when compared with the control condition. The diamond at the base of the plot represents the combined effect (Standardized Mean Difference) with 95% CIs.

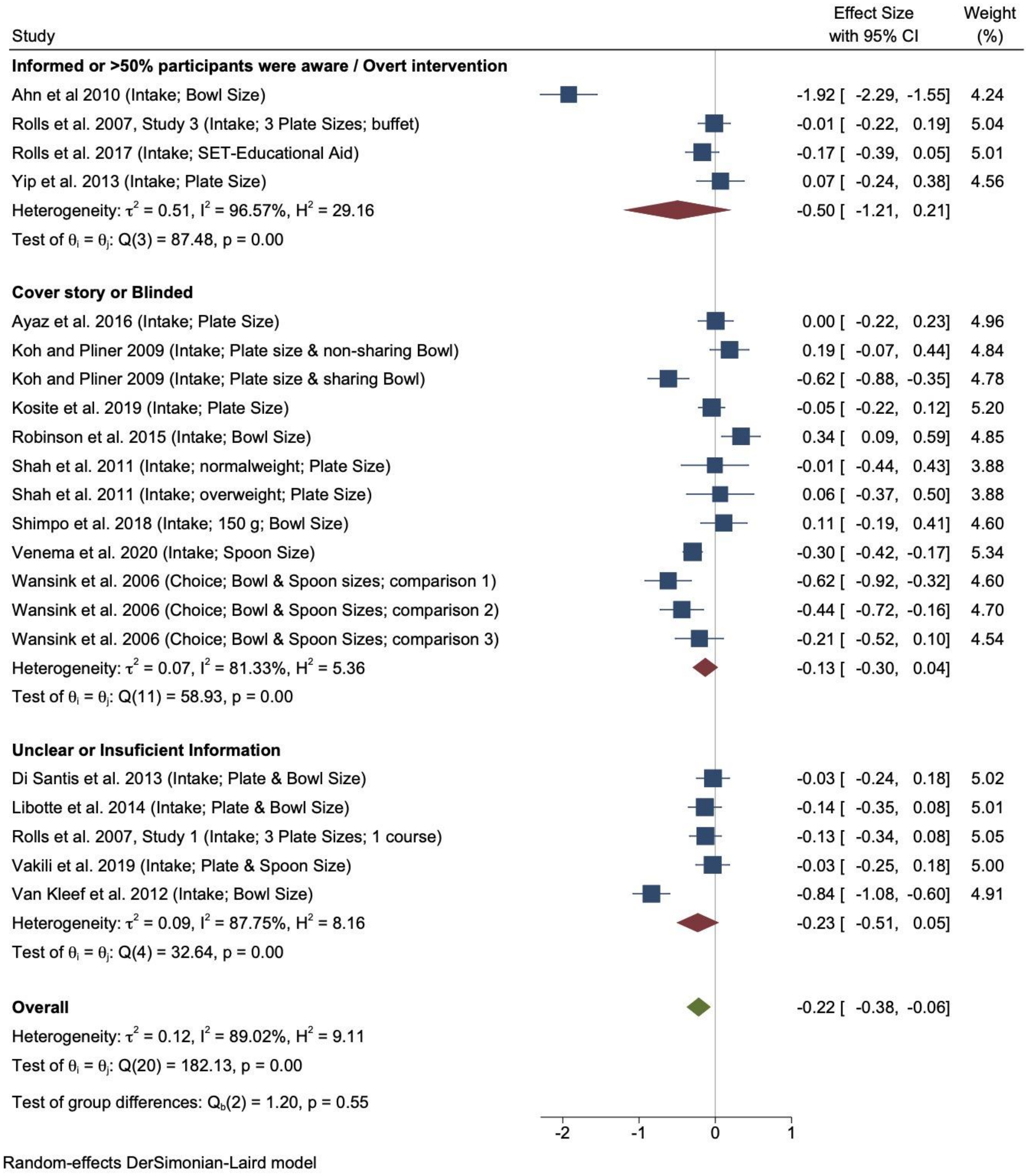

Figure S3. Sensitivity analysis for studies examining the impact of portion control tools on portion size intake by purpose awareness excluding data from Wansink et al. (2006)[11].

Forest plot for the analysis of all comparisons examining the effects of portion control tools on portion size intake (consumed amount) by study purpose awareness using a random-effects meta-analysis. Contributing comparisons are represented by a filled square with horizontal lines, where the area of the square depicts the contribution of the study to the full analysis, and the horizontal lines indicate the 95% CIs for each study. Studies displaced to the left of a 0 line demonstrate a finding in favour of the portion control tool to reduce food intake, whereas those to the right demonstrate a finding in favour of the portion control tool to increase consumed amounts when compared with the control condition. The diamond at the base of the plot represents the combined effect (Standardized Mean Difference) with 95% CIs.

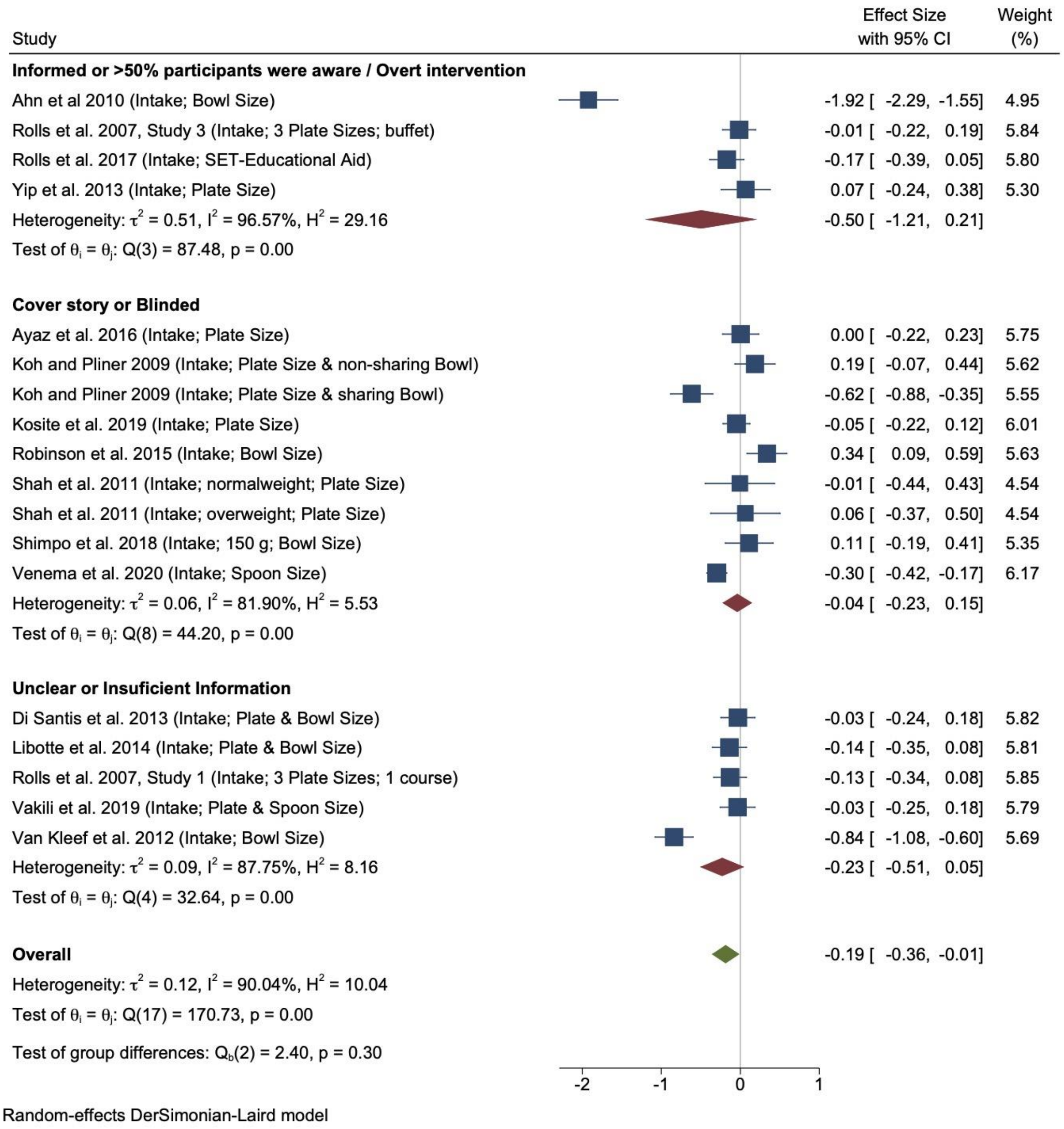

[11] Wansink B, van Ittersum K, Painter JE. Ice cream illusions bowls, spoons, and self-served portion sizes. Am J Prev Med. 2006;31(3):240–3.

Figure S4. Impact of portion control tools on portion size intake by strategy (e.g. portion tool alone vs. portion tool plus dietetic counselling).

Forest plot for the analysis of all comparisons examining the effects of portion control tools on portion size intake (consumed amount) by strategy using a random-effects meta-analysis. Contributing comparisons are represented by a filled square with horizontal lines, where the area of the square depicts the contribution of the study to the full analysis, and the horizontal lines indicate the 95% CIs for each study. Studies displaced to the left of a 0 line demonstrate a finding in favour of the portion control tool to reduce food intake, whereas those to the right demonstrate a finding in favour of the portion control tool to increase consumed amounts when compared with the control condition. The diamond at the base of the plot represents the combined effect (Standardized Mean Difference) with 95% CIs.

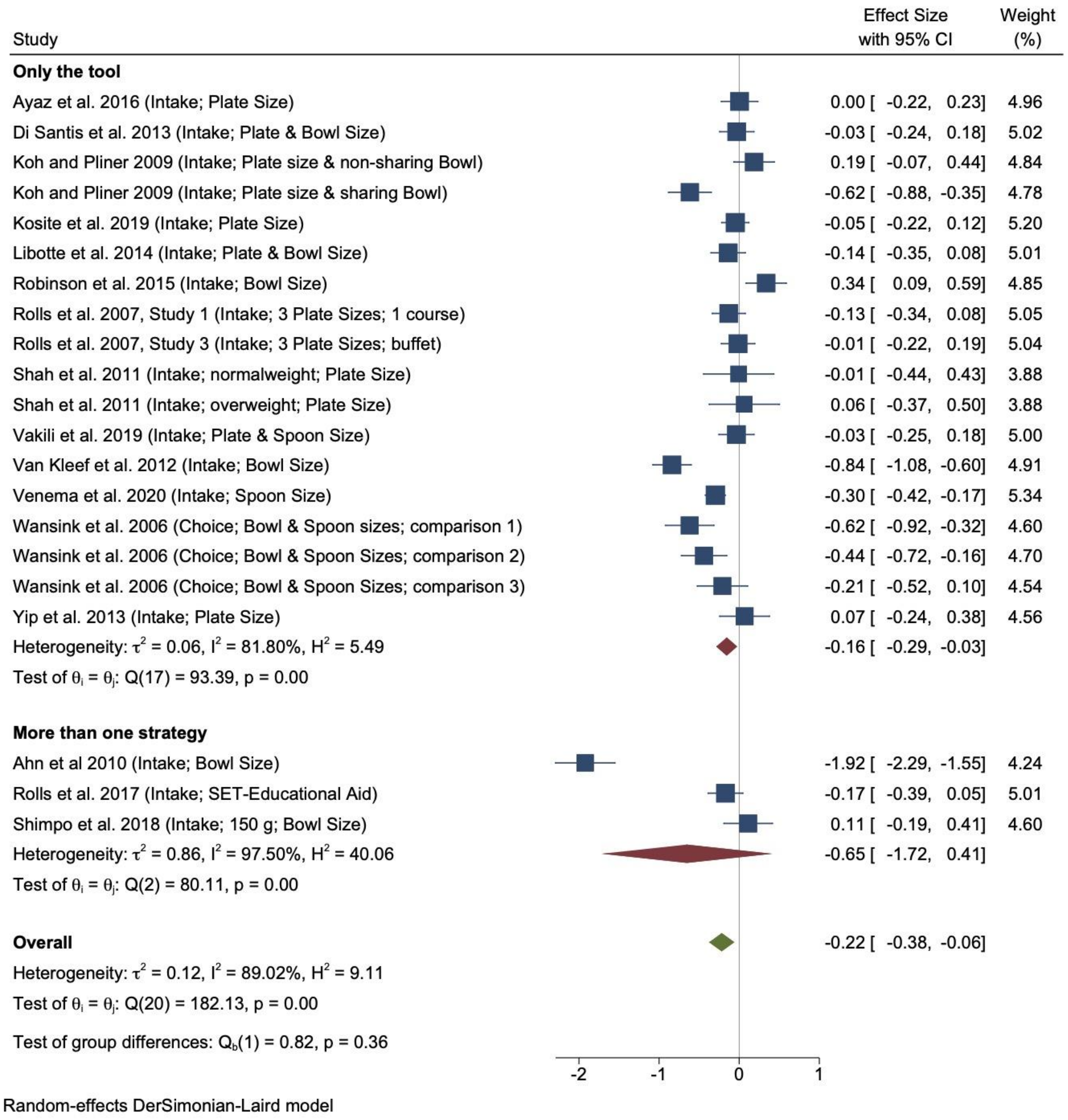

Figure S5. Sensitivity analysis for studies examining the impact of portion control tools on portion size intake by strategy excluding data from Wansink et al. (2006)[12].

Forest plot for the analysis of all comparisons examining the effects of portion control tools on portion size intake (consumed amount) by strategy using a random-effects meta-analysis. Contributing comparisons are represented by a filled square with horizontal lines, where the area of the square depicts the contribution of the study to the full analysis, and the horizontal lines indicate the 95% CIs for each study. Studies displaced to the left of a 0 line demonstrate a finding in favour of the portion control tool to reduce food intake, whereas those to the right demonstrate a finding in favour of the portion control tool to increase consumed amounts when compared with the control condition. The diamond at the base of the plot represents the combined effect (Standardized Mean Difference) with 95% CIs.

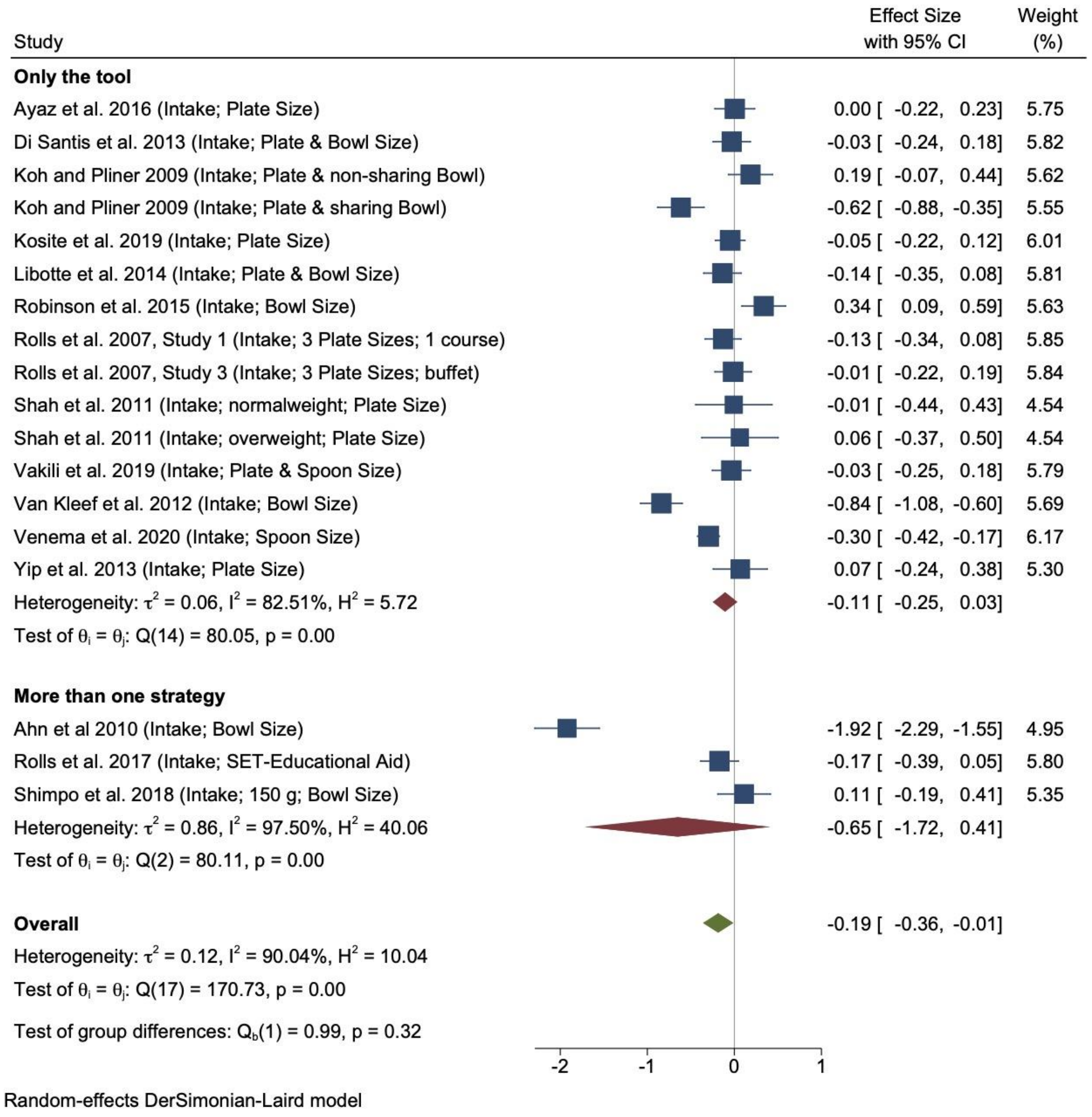

[12] Wansink B, van Ittersum K, Painter JE. Ice cream illusions bowls, spoons, and self-served portion sizes. Am J Prev Med. 2006;31(3):240–3.

Figure S6. Sensitivity analysis for studies examining the impact of portion control tools on portion size intake by tool type using the large rice portion size in Shimpo & Akamatsu (2018)[13] i.e. 250 g.

Forest plot for the analysis of all comparisons examining the effects of portion control tools on portion size intake (consumed amount) by tool type a random-effects meta-analysis. Contributing comparisons are represented by a filled square with horizontal lines, where the area of the square depicts the contribution of the study to the full analysis, and the horizontal lines indicate the 95% CIs for each study. Studies displaced to the left of a 0 line demonstrate a finding in favour of the portion control tool to reduce food intake, whereas those to the right demonstrate a finding in favour of the portion control tool to increase consumed amounts when compared with the control condition. The diamond at the base of the plot represents the combined effect (Standardized Mean Difference) with 95% CIs.

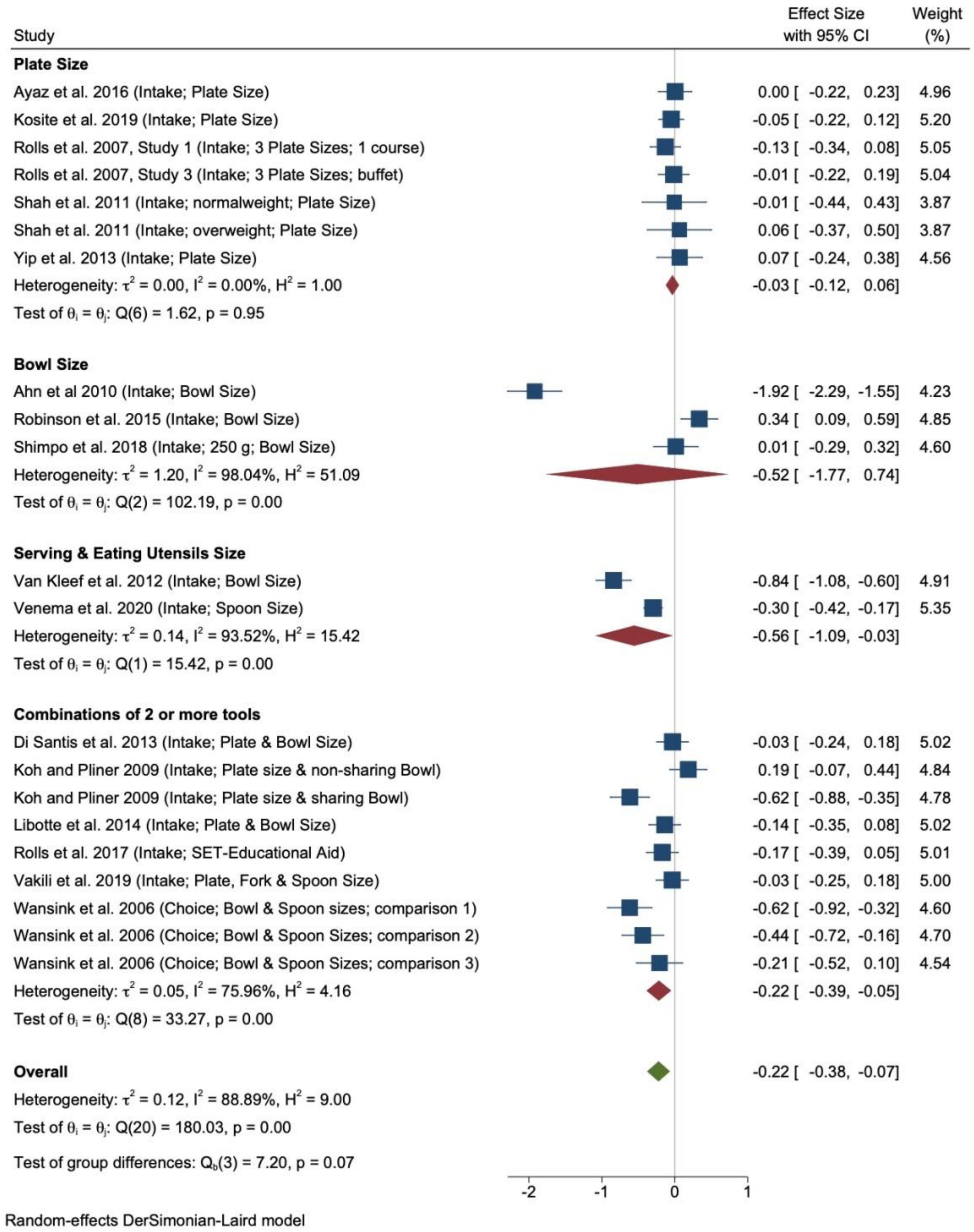

[13] Shimpo M, Akamatsu R. The effects of bowl size and portion size on food intake and fullness ratings in a sample of Japanese men. Public Health Nutr. 2018;21(17):3216–22.

Figure S7. Impact of portion control tools on weight status by study duration (3 months outcome).

Forest plot for the analysis of all comparisons examining the effects of portion control tools on weight status (weight loss or BMI change) at 3 months random-effects meta-analysis. Contributing comparisons are represented by a filled square with horizontal lines, where the area of the square depicts the contribution of the study to the full analysis, and the horizontal lines indicate the 95% CIs for each study. Studies displaced to the left of a 0 line demonstrate a finding in favour of the portion control tool helping to reduce body weight, whereas those to the right demonstrate a finding in favour of the tool to promote a higher BMI or weight gain, when compared with the control condition. The diamond at the base of the plot represents the combined effect (Standardized Mean Difference) with 95% CIs.

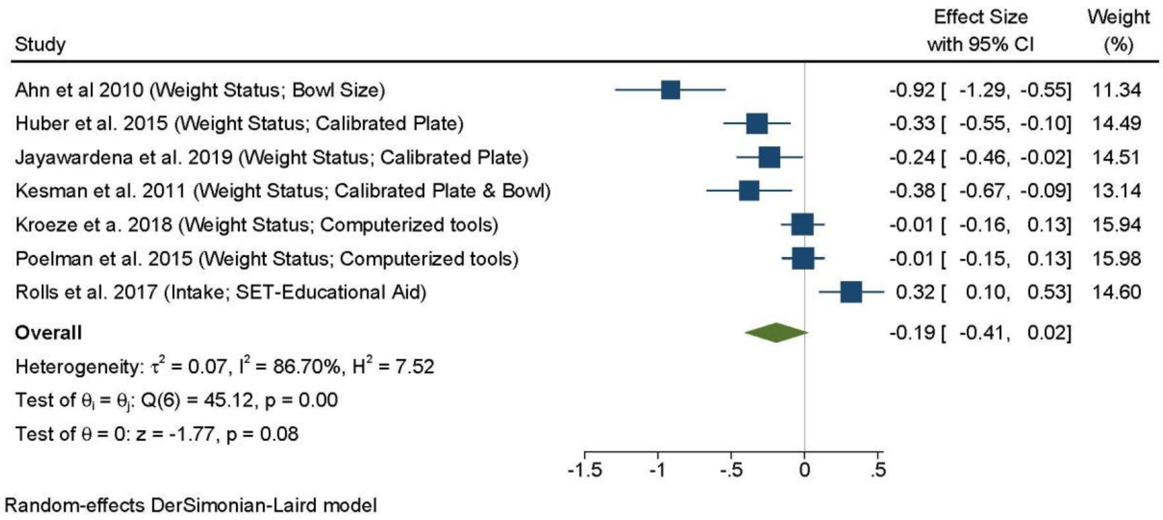

Figure S8. Impact of portion control tools on weight status by study duration (6 months outcome).

Forest plot for the analysis of all comparisons examining the effects of portion control tools on weight status (weight loss or BMI change) at 6 months or after random-effects meta-analysis. Contributing comparisons are represented by a filled square with horizontal lines, where the area of the square depicts the contribution of the study to the full analysis, and the horizontal lines indicate the 95% CIs for each study. Studies displaced to the left of a 0 line demonstrate a finding in favour of the portion control tool helping to reduce body weight, whereas those to the right demonstrate a finding in favour of the tool to promote a higher BMI or weight gain, when compared with the control condition. The diamond at the base of the plot represents the combined effect (Standardized Mean Difference) with 95% CIs.

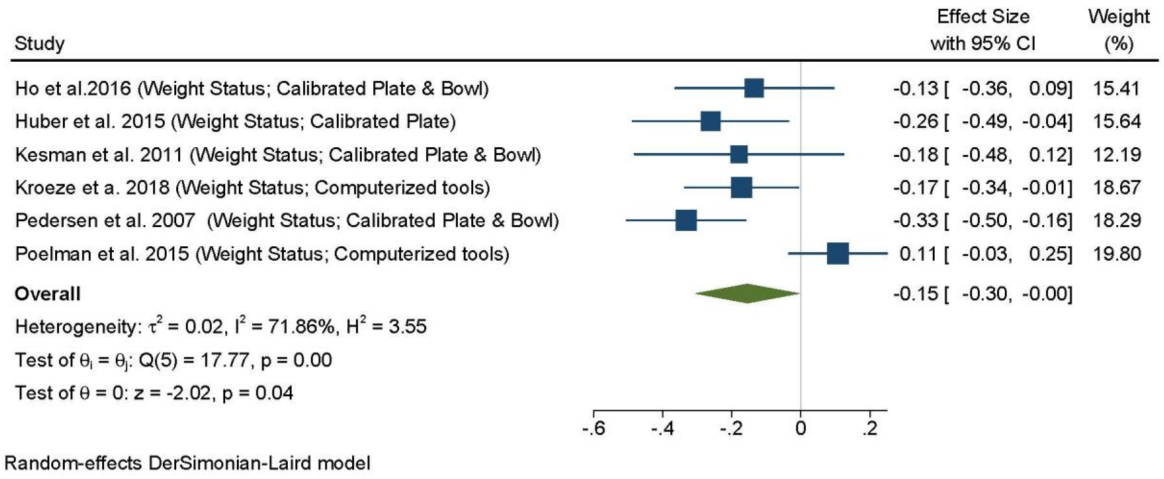

Figure S9. Funnel plots for studies exploring weight status using random-effects (left) and fixed-effects (right) meta-analysis. The number of studies included for each plot is of n=9.

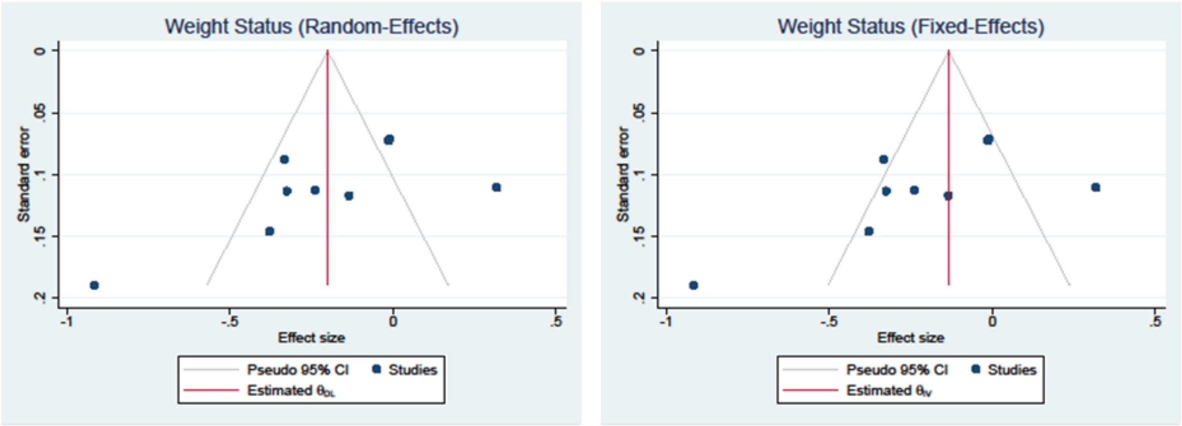

Supplement: Supplementary file 1 [file nutrients-13-01978-s001.zip › nutrients-1184196-SI.pdf]
